# Potent neutralizing antibodies elicited by dengue vaccine in rhesus macaque target diverse epitopes

**DOI:** 10.1371/journal.ppat.1007716

**Published:** 2019-06-06

**Authors:** Leike Li, Weixu Meng, Melanie Horton, Daniel R. DiStefano, Elizabeth A. Thoryk, Jennifer M. Pfaff, Qihui Wang, Georgina T. Salazar, Trevor Barnes, Benjamin J. Doranz, Andrew J. Bett, Danilo R. Casimiro, Kalpit A. Vora, Zhiqiang An, Ningyan Zhang

**Affiliations:** 1 Texas Therapeutics Institute, Brown Foundation Institute of Molecular Medicine, University of Texas Health Science Center at Houston, Houston, Texas, United States of America; 2 Department of Infectious Diseases and Vaccines Research, Merck Research Laboratories, Merck and Co. Inc., Kenilworth, New Jersey, United States of America; 3 Integral Molecular, Philadelphia, Pennsylvania, United States of America; 4 CAS Key Laboratory of Microbial Physiological and Metabolic Engineering, Institute of Microbiology, Chinese Academy of Sciences, Beijing, China; Purdue University, UNITED STATES

## Abstract

There is still no safe and effective vaccine against dengue virus infection. Epidemics of dengue virus infection are increasingly a threat to human health around the world. Antibodies generated in response to dengue infection have been shown to impact disease development and effectiveness of dengue vaccine. In this study, we investigated monoclonal antibody responses to an experimental dengue vaccine in rhesus macaques. Variable regions of both heavy chain (VH) and light chain (VL) were cloned from single antibody-secreting B cells. A total of 780 monoclonal antibodies (mAbs) composed of paired VH and VL were characterized. Results show that the vaccination induces mAbs with diverse germline sequences and a wide range of binding affinities. Six potent neutralizing mAbs were identified among 130 dengue envelope protein binders. Critical amino acids for each neutralizing antibody binding to the dengue envelope protein were identified by alanine scanning of mutant libraries. Diverse epitopes were identified, including epitopes on the lateral ridge of DIII, the I-III hinge, the bc loop adjacent to the fusion loop of DII, and the β-strands and loops of DI. Significantly, one of the neutralizing mAbs has a previously unknown epitope in DII at the interface of the envelope and membrane protein and is capable of neutralizing all four dengue serotypes. Taken together, the results of this study not only provide preclinical validation for the tested experimental vaccine, but also shed light on a potential application of the rhesus macaque model for better dengue vaccine evaluation and design of vaccines and immunization strategies.

## Author summary

Dengue virus (DENV) is a leading cause of human illness in the tropics and subtropics, with about 40% of the world’s population living in areas at risk for infection. There are four DENV serotypes. Patients who have previously been infected by one dengue serotype may develop more severe symptoms such as bleeding and endothelial leakage upon secondary infection with another dengue serotype. This study reports the extensive cloning and analysis of 780 monoclonal antibodies (mAbs) from single B cells of rhesus macaques after immunization with an experimental dengue vaccine. We identified a panel of potent neutralizing mAbs with diverse epitopes on the DENV envelope protein. Antibodies in this panel were found to bind to the lateral ridge of DIII, the I-III hinge, the bc loop adjacent to the fusion loop of DII, and the β-strands and the loops of DI. We also isolated one mAb (d448) that can neutralize all four dengue serotypes and binds to a novel epitope at the interface of the DENV envelope and membrane proteins. Further investigation of these neutralizing monoclonal antibodies is warranted for better vaccine efficacy evaluation and vaccine design.

## Introduction

Dengue virus (DENV), a mosquito-borne flavivirus, causes an estimated 390 million infections annually and presents a formidable burden on the global healthcare system [1]. DENV infection can result in common dengue fever (DF) and more severe illnesses such as dengue hemorrhagic fever (DHF) and dengue shock syndrome (DSS). DHF and DSS are characterized by increased vascular permeability, hemorrhagic manifestations, and plasma leakage-induced shock that can lead to death. Approximately 40% of the world’s population lives in dengue-endemic regions, so the spread of DENV infection and threat of severe disease remain a significant challenge to global human health [2,3].

DENV has four serologically distinct subtypes, DENV1, DENV2, DENV3, and DENV4, each with an RNA genome of 10.7 kb that is translated into three structural proteins, Capsid (C), Envelope (E), preMembrane (prM), and seven nonstructural proteins (NS1, NS2A, NS2B, NS3, NS4A, NS4B and NS5) [4]. Dengue virus exhibits substantial genetic diversity, with approximately 30%-40% amino acid sequence divergence between serotypes [4]. Immunity induced by primary infection of DENV is effective in protection against subsequent infection by the homologous viral serotype. However, when secondary infection by a different serotype occurs, pre-existing immunity to one serotype of DENV can be an adverse factor causing life-threatening illness such as DHF or DSS [5–7]. Thus, it is critical for a DENV vaccine to induce a balanced immune response or elicit broadly neutralizing antibodies against all four serotypes [8,9].

The licensed dengue vaccine, Dengvaxia, has limited efficacy in protecting against DENV infection. This efficacy depends on the serotype of the infecting virus and the immune status at the time of vaccination [10,11]. Unexpectedly, the vaccine has lower efficiency in subjects who were DENV seronegative than in subjects who were DENV seropositive prior to vaccination [12,13]. In Phase 3 trials, the vaccine showed 35–80% protection against infection by all DENV serotypes. However, this efficacy is not consistent with the anti-DENV titer as assessed in the clinical trial. In this trial, over 90% of the subjects showed serum neutralization activity against DENV [14,15]. This result indicates that titer is not sufficient for dengue vaccine evaluation. Because of the complexity of viral antigens involved and the polyclonal nature of immune sera, other factors may influence vaccine efficacy. An alternative approach to estimate the efficacy of a dengue vaccine in clinical trials is through a better understanding of the monoclonal antibodies (mAbs) induced by the vaccine. Antibody responses can be measured accurately, in both qualitative and quantitative terms, and the epitopes can be mapped for potent neutralizing antibodies. Therefore, vaccine efficacy could be evaluated by serum titer, monoclonal antibody response, and epitopes of neutralizing antibodies.

Non-human primates (NHP) such as rhesus macaques can develop viremia upon DENV infection and have a qualitative immune response similar to that in humans [16]. Therefore, they are often used to evaluate dengue vaccine candidates in preclinical trials. Induction of a humoral immune response by vaccination has been shown to induce protective immunity against dengue infection [17,18]. We have developed a dengue subunit tetravalent vaccine comprising truncated E proteins, 80% of the N-terminal of the E protein from all four DENV serotypes (named DEN-80E) [19,20]. It was shown that a prime-boost strategy (live attenuated virus prime, DEN-80E boost) in rhesus macaque induced neutralization titers to all four DENV serotypes that were higher than those induced by a prime-boost regime using only tetravalent virus vaccine [21]. To better evaluate the efficacy of the vaccine, we developed a robust method to clone monoclonal antibodies from the antibody-secreting cells of rhesus macaque after vaccination. By characterizing these antibodies, we are able to identify indicators of vaccine efficacy [22].

The DENV E protein is the dominant antigenic site of DENV neutralizing antibody. It forms dimeric structures that anchor on the M protein through hydrophobic interaction on the mature virus particle [23,24]. Each E protein contains three domains: DI, DII, and DIII [25]. DI participates in the conformational changes of the virus particle [26,27]. DII interacts with the endosome to lead fusion of the virus after entry using the hydrophobic fusion loop [28]. DIII is involved in receptor binding [29,30]. The prM protein forms a heterodimer with E protein to prevent premature fusion [31]. During virus assembly in the endoplasmic reticulum (ER), each set of three prM-E heterodimers form “spikes” on the virion surface. The virus is then transported through the secretory pathway. In the trans-Golgi, the low-pH environment induces the rearrangement of prM-E heterodimers into a flattened conformation. Then the prM protein on immature virions is cleaved by furin or furin-like protease to produce mature virions [29]. However, the cleavage process is incomplete, leading to a mixture of virus particles in different states of maturity [32]. The diversity of virus structures influences epitope accessibility and thereby affects the neutralizing activity of the antibodies targeting different epitopes [33].

Comprehensive studies have identified both serotype-specific and serotype-cross-reactive epitopes in all three domains of the E protein, recognized by various neutralizing mAbs [34,35]. The mouse antibodies to DIII exhibit potent neutralizing activity. Their neutralization capacity and specificity vary between DENV serotypes. [36–38]. The lateral ridge (loops between the β-sheets) of DIII, fully accessible on the intact virion, is an epitope hotspot for mouse neutralizing antibodies. It is also a neutralizing site for other flaviviruses, such as Zika and West Nile virus [39,40]. Besides the lateral ridge, mouse antibody targeting the A strand of DIII, has demonstrated strong cross-serotype neutralizing activity, drawing attention to potential immunotherapy applications. DIII is also the antigenic region of the human antibody response. The antibodies isolated from human patients have been shown to bind the lateral ridge and the stands of DIII [41]. However, the proportion of DIII-targeting human neutralizing antibodies is low in dengue-positive human sera [42]. Humans tend to produce antibodies that bind to epitopes in domains other than DIII [43]. In general, the fusion loop of DII represents a dominant epitope, and the antibodies specific for this loop are weak and tend to have cross-reactive neutralizing activity against different flavivirus infections [44,45]. On the other hand, the bc loop adjacent to the fusion loop in DII is an epitope of a highly potent human antibody neutralizing all four DENV serotypes [46]. It has also been reported that mouse antibodies can block virus membrane fusion by targeting the fusion loop and bc loop simultaneously [47,48]. These examples highlight the importance of the loop adjacent to the fusion loop in inhibiting DENV infection. As for the DI-binding mAbs, the lateral ridge of DI and the hinge region are the epitopes targeted by neutralizing antibodies from mouse, chimpanzee, and human [49,50]. Generally, the DI antibodies neutralize the virus by blocking the conformational change of the E protein during membrane fusion [50]. Another group of antibodies targets the quaternary epitope whose critical residues lie at the domain interface or across different domains. Recently, a new class of potent neutralizing human mAbs was reported, only targeting the E-dimer on the intact virus surface [51,52]. These antibodies are E-dimer dependent, locking the prefusion structure of E protein, preventing the conformational change necessary for virus fusion. They are the dominant neutralizing antibodies in human patients. However, a comprehensive understanding of the DENV epitopes targeted by rhesus macaque antibodies has not been described.

In this study, we investigated antibody responses to an experimental dengue vaccine in rhesus macaques. We cloned and characterized a large panel of mAbs (780 antibodies) from single antibody-secreting B cells (ASCs) using a high-throughput cloning protocol that we reported previously [22]. Six potent neutralizing antibodies were identified. Their binding epitopes were mapped using an alanine-scan mutation library across dengue prM/E [53]. We isolated antibodies with binding epitopes similar to those previously reported [36,46,50,54]. This collection of antibodies exhibited a diverse epitope pattern, with binding sites on the lateral ridge of DIII (d182, d511, and d628), the I-III hinge (d182), the bc loop adjacent to the fusion loop in DII (d559), and the β-strands and loops of DI (d462). More significantly, we identified a broadly neutralizing antibody (d448) recognizing an epitope never previously reported in DII, at the interface of the E and M proteins. Discovery of these antibodies with diverse epitopes will aid the design of more effective vaccines and therapeutic antibodies against dengue infection.

## Results

### Diverse antibody response to dengue vaccination in rhesus macaques

**Fig 1:**
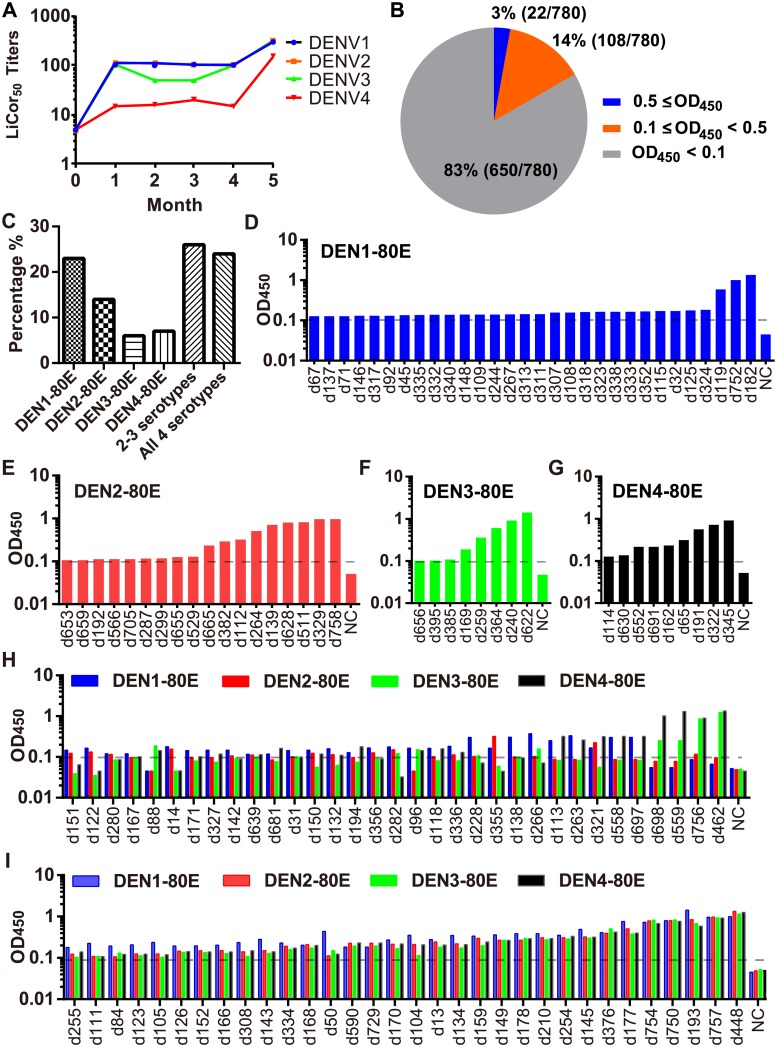
Characterization of dengue targeting monoclonal antibodies from antibody-secreting B cells of the rhesus macaque. (A) The longitudinal geometric mean neutralization titers of the rhesus sera for DENV1, DENV2, DENV3, and DENV4, respectively. The LiCor_50_ Titers indicate the dilution rate of the sera maintains 50% of the neutralizing activity. (B) The pie chart shows the distribution of antibodies that bound DEN-80E strongly, weakly, or not at all, as determined by ELISA screening. The antibodies were considered to be strong binders when OD_450_ was above or equal to 0.5 (3%), weak binders when OD_450_ was between 0.1 and 0.5 (14%), and non-binders when OD_450_ was below 0.1 (83%). (C) Percentage of binders specific for single and multiple dengue serotypes among antibodies binding DEN-80E. (D-I) Reactivity for DEN-80E binding in ELISA screening for antibodies binding DEN-80E of DENV1 (D), DENV2 (E), DENV3 (F), DENV4 (G), 2 or 3 dengue serotypes (H), all four dengue serotypes (I). The X-axis indicates individual antibodies, the black dotted lines indicate the cut-off OD_450_ of 0.1, and NC means negative control.

The experimental vaccine of this study uses live attenuated viruses (LAV) for priming and tetravalent recombinant E proteins (DEN-80E) for boosting [21,22]. The immunization schedules and workflow for antibody analysis are outlined in S1 Fig. Briefly, a total of 12 Rhesus macaques received a primer injection of the LAV subcutaneously (S.C.) at week 0, followed by a boost of tetravalent DEN-80E intramuscularly (I.M.) at week 16 (month 4). This vaccine has shown to elicit robust DENV neutralizing antibody titers in rhesus macaques (Fig 1A) [19,55]. To analyze the antibody response to the vaccine, seven days after the booster dose, we isolated monoclonal antibodies from single ASCs by fluorescence-activated cell sorting (FACS) from peripheral blood mononuclear cells (PBMCs) of 12 vaccinated rhesus macaques. We pooled the PBMCs together for the ASCs sorting, and cloned a total of 780 antibodies from more than a thousand ASCs collected using a method that we reported previously [22]. The single ASCs were isolated by FACS from PBMCs into 96-well plates (one cell/well). The natively paired heavy and light variable regions from single B cells were cloned using a direct cDNA synthesis and nested-PCR amplification strategy. All paired antibodies were cloned into a framework of human IgG1 and expressed as macaque-human chimeric antibodies in HEK293-F cells for purification and downstream analysis. The initial screening of E protein binders was performed with supernatants of antibody expression cultures to eliminate non-binding antibodies. Positive binding antibodies (mAbs) were defined as those with an OD_450_ ≥ 0.1 as assessed by ELISA in the primary screen. Among the cloned 780 mAbs, 130 (17%) of the supernatant antibodies were DEN-80E binders: 106 (14%) mAbs with OD450 in the range of 0.1 to 0.5 were defined as weak binders; 22 (3%) antibodies with OD450 ≥ 0.5 were considered to be strong binders (Fig 1B). Among the 130 binding mAbs, there were different types of cross-recognition of the four serotypes of DEN-80E: single serotype, two or three serotypes, and all four serotypes (Fig 1C). About 50% of the DENV binding mAbs were specific to only one serotype of dengue: 30 mAbs were specific to DEN1-80E (Fig 1D); 18 mAbs to DEN2-80E (Fig 1E); 8 mAbs to DEN3-80E (Fig 1F); and 9 mAbs to DEN4-80E (Fig 1G). A total of 33 mAbs showed binding to DEN-80E proteins from two or three dengue serotypes (Fig 1H). More significantly, 32 cloned mAbs had cross-reactivity to all four dengue serotypes (Fig 1I). In summary, diverse antibodies recognizing complex epitopes formed the macaque antibody response to the vaccination.

Positive binding mAbs were purified for further evaluation, most of the cloned antibodies showed low-affinity binding. Eighteen cloned antibodies showed a half-maximal binding concentration (EC_50_) in the sub-microgram to picogram range for envelope proteins from one or more DENV serotypes (Table 1, S2 Fig).

**Table 1:** Binding activity of DENV-specific mAbs.

mAbs	EC_50_ (μg/ml) to indicated dengue envelope protein (DEN-80E)^a^			
	DEN1-80E	DEN2-80E	DEN3-80E	DEN4-80E
d13	7.30	-	-	-
d50	-	6.06	-	>
d104	-	0.75	-	3.89
d145	-	9.60	-	-
d149	-	9.73	-	-
d177	3.26	-	-	-
d552	-	-	-	3.30
d558	1.75	-	-	1.43
d590	-	0.50	-	9.98
d665	0.40	-	-	-
d376	0.28	0.24	0.05	0.21
d182	0.0017	-	-	-
d511	-	0.0018	-	-
d628	-	0.0019	-	-
d622	-	-	0.0034	-
d559	-	-	-	0.0032
d462	-	-	0.11	0.0031
d448	0.0221	0.0017	0.0018	0.076

^***a***^The EC_50_ values are estimated for the concentration (μg/ml) of antibody achieved at half-maximal binding to the DEN-80E proteins. The “-” indicates no binding. The “>” indicates the EC_50_ is above 10 μg/ml.

### Neutralization activity of dengue-specific antibodies

**Fig 2:**
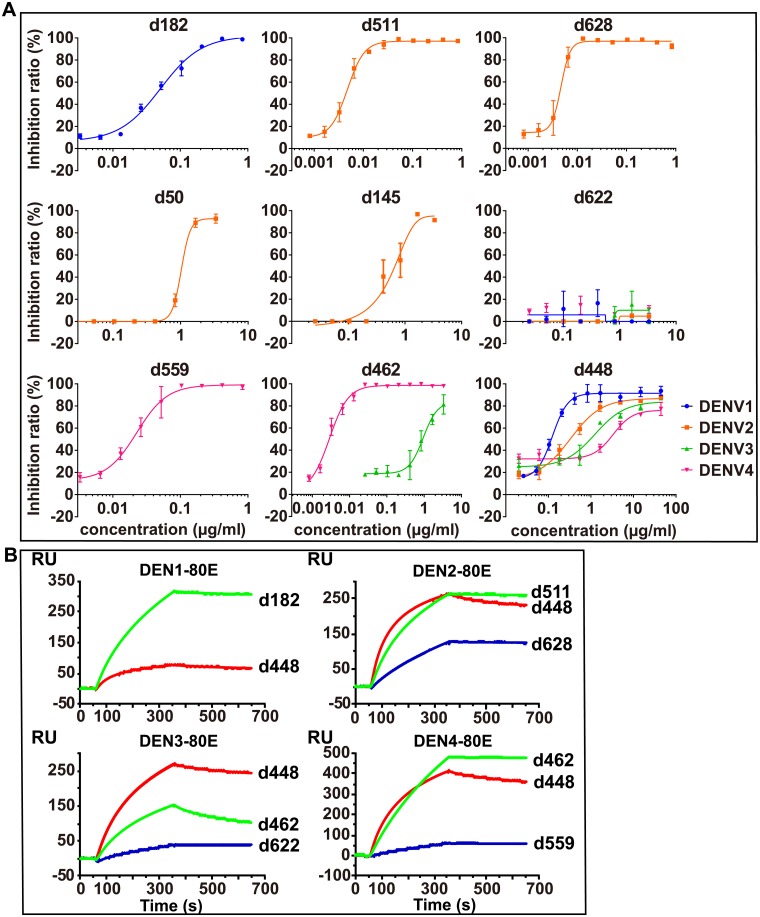
Neutralization activity and kinetic analysis of the potent neutralizing antibodies. (A) Neutralization activity of the indicated antibodies to DENV. The virus was mixed with 5-fold serial dilutions of antibodies. Then neutralization activity was evaluated by micro-neutralization assay in duplicates. Data are represented as mean ± SEM. The curves are fitted by the Response—Variable slope (four parameters) model. (B) Binding kinetics of selected antibodies on immobilized DEN-80E. The graphs show kinetic binding of six potent neutralizing antibodies at the concentration of 25 nM to immobilized antigens DEN1-80E, DEN2-80E, DEN3-80E, and DEN4-80E as indicated.

Next, using purified antibodies, we screened the potent binders for viral neutralizing activity. All four DENV serotypes were evaluated. NT_50_ (antibody concentration required to produce 50% viral neutralization) is estimated based on the antibody titration curves (Fig 2A) using a 4-parameter fitting model. NT_50_ values are listed in Table 2.

**Table 2:** Neutralization activity for the selected DENV mAbs.

mAbs	NT_50_ (μg/ml) against indicated DENV serotype^a^			
	DENV1	DENV2	DENV3	DENV4
d182	0.05	-	-	-
d511	-	0.0047	-	-
d628	-	0.0046	-	-
d50	-	1.02	-	-
d145	-	0.59	-	-
d622	-	-	-	-
d559	-	-	-	0.0226
d462	-	-	0.9476	0.0029
d448	0.13	0.33	1.25	3.33

^***a***^The concentration at which half-maximal inhibition of the dengue virus infection (NT_50_) is achieved is shown for each dengue virus serotype. The “-” indicates no inhibition of DENV infection.

Antibodies specific to only one DENV serotype demonstrated higher potency and binding affinity. As shown in Table 2, d182 neutralized only DENV1 with an NT_50_ of 0.05 μg/ml. Antibodies d511 and d628 neutralized only DENV2. Antibody d511 neutralized DENV2 with an NT_50_ of 0.0047. Antibody d628 neutralized DENV2 with an NT_50_ of 0.0046 μg/ml. Two other mAbs, d50 and d145, inhibit DENV2 infection at higher concentrations, with NT_50_ of 1.02 μg/ml and 0.59 μg/ml, respectively. Notably, the DENV3 specific antibody d622 demonstrated no inhibition of DENV infection, despite its high binding affinity to DEN3-80E (Table 3). Antibody d559 neutralized only DENV4 with an NT_50_ of 0.0226 μg/ml. The cross-reactive antibodies had lower binding affinities and neutralizing potency than did single-serotype specific mAbs. One antibody, d462, showed neutralizing activity against both DENV3 and 4 with DENV3 at NT_50_ 0.9476 μg/ml and DENV4 at NT_50_ 0.0029 μg/ml. The antibody d448 showed cross-neutralizing potency against all four serotypes with NT50 for DENV1 at 0.13 μg/ml, NT50 for DENV2 at 0.33 μg/ml, NT50 for DENV3 at 1.25 μg/ml, and NT50 for DENV4 at 3.33 μg/ml (Table 2).

**Table 3:** Kinetic binding constants of the DENV neutralizing mAbs.

mAbs	Serotypes	Kinetic binding constants ^a^		
		*ka* (1/Ms)	*kd* (1/s)	*K*_*D*_ (nM)
d182	DEN1-80E	2.12E+05	8.63E-05	0.408
d511	DEN2-80E	1.82E+05	1.90E-05	0.104
d628	DEN2-80E	1.09E+05	9.36E-05	0.857
d622	DEN3-80E	1.67E+04	2.28E-07	0.014
d559	DEN4-80E	3.00E+04	4.79E-07	0.016
d462	DEN3-80E	1.28E+05	1.45E-03	11.300
	DEN4-80E	1.16E+05	3.55E-07	0.003
d448	DEN1-80E	4.89E+05	3.90E-04	0.798
	DEN2-80E	4.96E+05	3.17E-04	0.640
	DEN3-80E	2.72E+05	2.74E-04	1.010
	DEN4-80E	6.13E+05	1.86E-04	0.304

^***a***^Association rate constants (*ka*), dissociation rate constants (*kd*), and the equilibrium dissociation constant (*K*_*D*_)

Next, using surface plasmon resonance (SPR), we determined kinetic binding constants of neutralizing mAbs, *ka* (on rate), *kd* (off rate), and *K*_*D*_ (equilibrium dissociation constant). The potent mAbs targeting specific DENV serotypes (d182, d511, d628, d622 and d559) demonstrated slow off rates (Table 3, Fig 2B, S3 Fig). The neutralizing activity of the mAbs is consistent with the binding affinity, except in the case of mAb d622.

Antibody d462 has similar on-rate (ka) for both DENV3 and DENV4. Interestingly, mAb d462 has a faster off-rate (kd) for DEN3-80E than for DEN4-80E and shows weaker neutralizing potency for DENV3 (NT50: 0.9476 μg/ml) than for DENV4 (NT50: 0.0029 μg/ml) (Table 3, Fig 2B). These results suggest that the slow off-rate (kd) of mAb d462 plays a key role in its DENV neutralizing activity.

### Epitope mapping of neutralizing mAbs

Identification of binding epitopes is crucial for understanding the mechanism of antibody function. We conducted epitope mapping for antibodies with strong neutralizing activities (d182, d511, d628, d559, d462, and d448) by screening a shotgun alanine scan mutagenesis library of dengue E proteins [53]. The critical residues for antibody bindings were identified within the indicated serotype (Table 4, S4 Fig).

**Table 4:** Epitope mapping of the selected neutralizing antibodies.

mAbs	Key amino acid residues	Serotypes
d182	V300, T329	DENV1
d511/d628	D329, K361	DENV2
d559	Y81, K83	DENV4
d462	V160, V173, D177	DENV4
d448	D215, P219, M237, Q256, G266	DENV4

**Fig 3:**
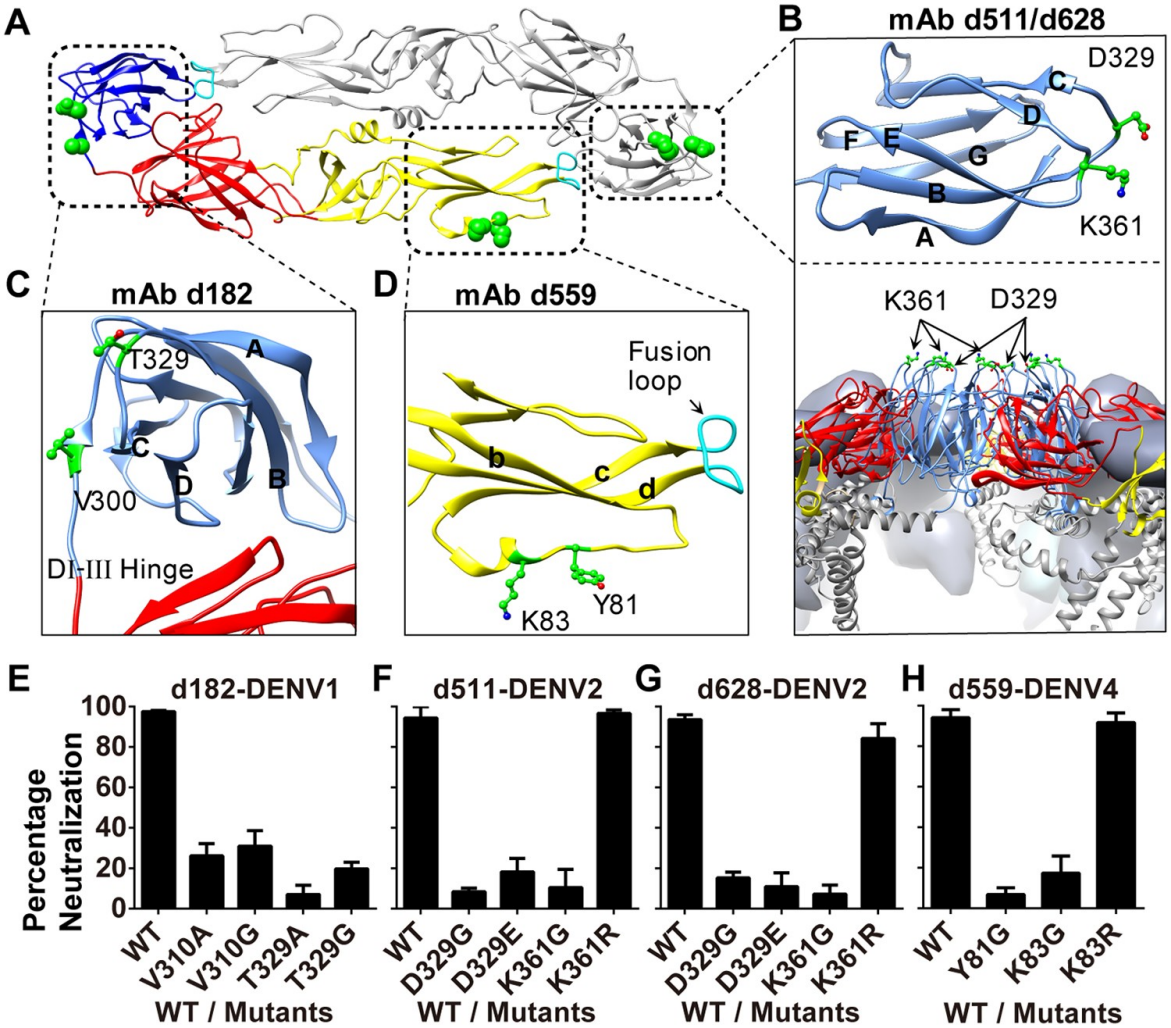
Critical residues for binding of dengue serotype-specific macaque antibodies. (A) Critical resides (green spheres) are mapped on the mature E protein dimer structure (PDB, 1UZG). Domain I is red, domain II yellow, domain III blue, and fusion loop cyan. The asymmetric monomer rotated 180 degrees is shown in gray. (B) The key residues of the d511/d628 epitope (shown on PDB, 1OK8). D329 (BC loop) and K361 (DE loop) are on the lateral ridge of DENV2 domain III, which is a reported epitope region of mouse anti-dengue neutralizing antibodies. Bottom panel: side view of the mature virus capsid indicates that the residues D329 and K361 display the side chains on the external surface of domain III (PDB, 3J27). (C) The key residues of the d182 epitope (PDB, 3G7T). T329 is in the BC loop of DENV1 domain III. V300 is in the DI-III hinge. (D) The key residues of the d559 epitope (PDB, 1OK8, modified). Y81 and K83 are in the bc loop of DENV4 domain II. Atoms colored red for O, blue for N. Molecular graphic images were produced using UCSF Chimera (http://www.rbvi.ucsf.edu/chimera). (E-H) Neutralization percentage of the indicated wild type (WT) DENV reporter virus particles, and its critical amino acid residue substitution mutants. Antibodies d182 (E), d511 (F), d628 (G), and d559 (H) were tested at the concentration of 3 μg/ml. The assays were performed in triplicates and data were analyzed using GraphPad Prism.

Amino acid residues key for binding of antibodies that have cross-reactive neutralizing activity fall into regions that are more conserved among the four DENV serotypes than regions containing residues key for binding of antibodies specific to a single serotype (S5 Fig). Antibodies d511 and d628 bind to the BC loop (D329) and DE loop (K361) in the lateral ridge of DENV2 DIII. Residues D329 and K361 are located at the tip of the loop turn, displaying their side chains on the external surface of the mature virus capsid (Fig 3A and 3B). The key residues D329 and K361 have been previously reported in mouse anti-DENV2 and DENV1 antibody studies [37,56]. Antibody d182 binds in the BC loop (T329) and I-III hinge region (V300) of DENV1 (Fig 3C). It has been demonstrated that mutations in the DI-III hinge impair dengue virus particle assembly in the cell [57]. It is worth noting that the epitopes of d182/d511/d628 are in the same BC loop of the lateral ridge (S5 Fig). This result suggests that the lateral ridge region is essential in dengue virus infection, as antibodies targeting the region possess potent DENV-neutralizing activities. Antibody d559 binds to the bc loop (Y81, K83) of DII on DENV4. This epitope adjacent to the fusion loop may contribute to blocking virus fusion with the endosomal membrane (Fig 3D). It has been reported that some highly potent cross-reactive human antibodies have epitopes in the bc loop [46]. However, antibody d559, with an epitope (Y81, K83) in the bc loop, showed binding only to DENV4. Notably, the two amino acids bound by d559 are not conserved among the DENV serotypes (S5 Fig). To confirm the epitopes, we generated DENV reporter virus particles (RVP), in which amino acid residues in the epitope were substituted with relative amino acids for their impact on neutralizing activity of the antibodies. The RVPs with a detectable titer were chosen for neutralization assay (S1 Table). As shown in Fig 3E, residue substitutions of V310, T329 abolished the neutralizing activity of d182, suggesting that these are key residues for interaction of the DENV1 E protein with the antibody. The mutations in DENV2-RVP (D329G/E, K361G), and DENV4-RVP (Y81G, K83G) inhibited neutralizing activity of the indicated antibodies (Fig 3F, 3G and 3H). These results validated the epitopes mapped by screening the shotgun alanine scan mutagenesis library of dengue E proteins. In addition, it is worthy to note that the Lysine substitution to Arginine in the DENV2 (K361R) and DENV4 (K83R) retain the neutralizing activity of the antibodies due to the similarity of the residues (Fig 3F, 3G and 3H).

**Fig 4:**
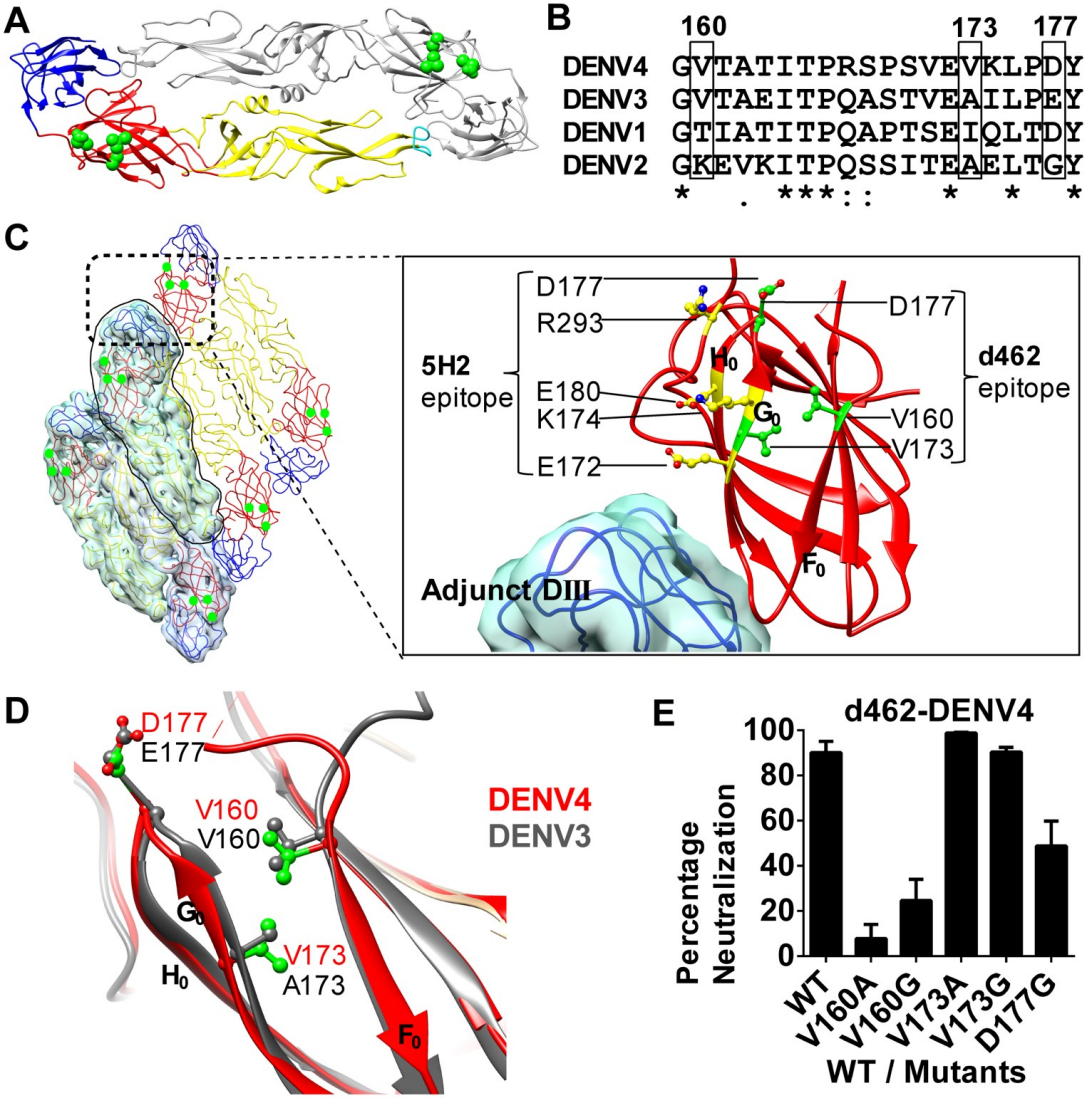
Critical residues for binding of cross-reactive antibody d462. The structural presentation of the critical residues of the epitope on DENV4 domain I. (A) Critical resides (green spheres) are mapped on both monomers of the mature E protein dimer structure (PDB, 1UZG). Domain I is red, domain II yellow, domain III blue and fusion loop cyan. The asymmetric monomer rotated 180 degrees is gray. (B) Sequence alignment of the epitope region on the E protein. Residues V160, V173 and D177 on DENV4 are not identical among different serotypes. However, they have similar chemical characteristics for DENV3 in position 173 (Valine compare to Alanine) and 177 (Aspartic acid compare to Glutamic acid). The NCBI accession numbers of the sequences are ACJ04226 (DENV1), AGS49173 (DENV2), AJA37731 (DENV3) and ACW82884 (DENV4) respectively. (C) Antibody d462 epitope on an E protein raft (PDB, 4CBF). The black line shows the boundary of a single asymmetric unit. The residues that interact with d462 are shown as green spheres. Enlarged view (right) shows the epitope region on F_0_, G_0_ β-strands, and the G_0_H_0_ loop on E protein domain I. The epitope of d462 (V160, V173, D177) is overlapped with that of the reported chimpanzee antibody 5H2 (E172, K174, D177, E180, R293). (D) Structural comparison of the epitope region on DENV4 (PDB, 3UC0) and DENV3 (PDB, 1UZG), indicating the structural similarity of the key residues. DENV4 is shown in red ribbon and DENV3 is shown in gray ribbon. The residues on DENV4 are shown as green sticks, on DENV3 as gray sticks. Atoms are colored red for O, blue for N. The analysis was performed using “Structural Comparison: Match -> Align” of UCSF chimera. Molecular graphic images were produced using the UCSF Chimera (http://www.rbvi.ucsf.edu/chimera). (E) Percentage neutralization of the DENV4 reporter virus particles, and its critical amino acid residue substitution mutants. Antibody d462 was tested at the concentration of 3 μg/ml. The assays were performed in triplicates and data were analyzed using GraphPad Prism.

Antibody d462, a cross-reactive antibody, recognized the F_0_ and G_0_ β-sheets and the G_0_H_0_ loop in DI of DENV4 (V160, V173, D177) (Fig 4A and 4B, S5 Fig). It has been reported that binding of chimpanzee antibody 5H2 inhibits the fusogenic conformational change in the adjacent monomer on the virus surface [50]. Antibody d462 has epitopes overlapping those of antibody 5H2 on DENV4 (Fig 4C). We measured the ability of each of the two mAbs to inhibit binding of the other to DEN4-80E using bio-layer interferometry. While d462 completely inhibited binding of 5H2 to DEN4-80E (S6A Fig), 5H2 also inhibited binding of d462 (S6B Fig), confirming that the two antibodies share overlapped epitopes on DEN4-80E. Antibody d462 may neutralize DENV4 by preventing fusion of the viral and endosomal membranes, as does the chimpanzee antibody 5H2. Although the key amino acids identified for the epitope of d462 on DENV4 are not identical among the four DENV serotypes, DENV3 and DENV4 share the same amino acid at position 160 (V), and very similar types of amino acids in two other locations (V vs. A at 173 and D vs. E in 177) (Fig 4C). The similarity in the amino acids at key positions may provide a structural basis for the cross-reactivity of d462 with DENV3 and DENV4 serotypes (Fig 4D). To validate the epitope in a functional assay, we measured the neutralizing activity of d462 to DENV4-RVPs with residue substitutions (V160A/G, V173A/G, D177G) (S1 Table). As shown in Fig 4E, V160 is the critical residue in the epitope, substitution of V160 to either alanine or glycine abolished the neutralizing activity of d462. In contrast, V173 and D177 are non-essential residues for the neutralizing activity of d462.

**Fig 5:**
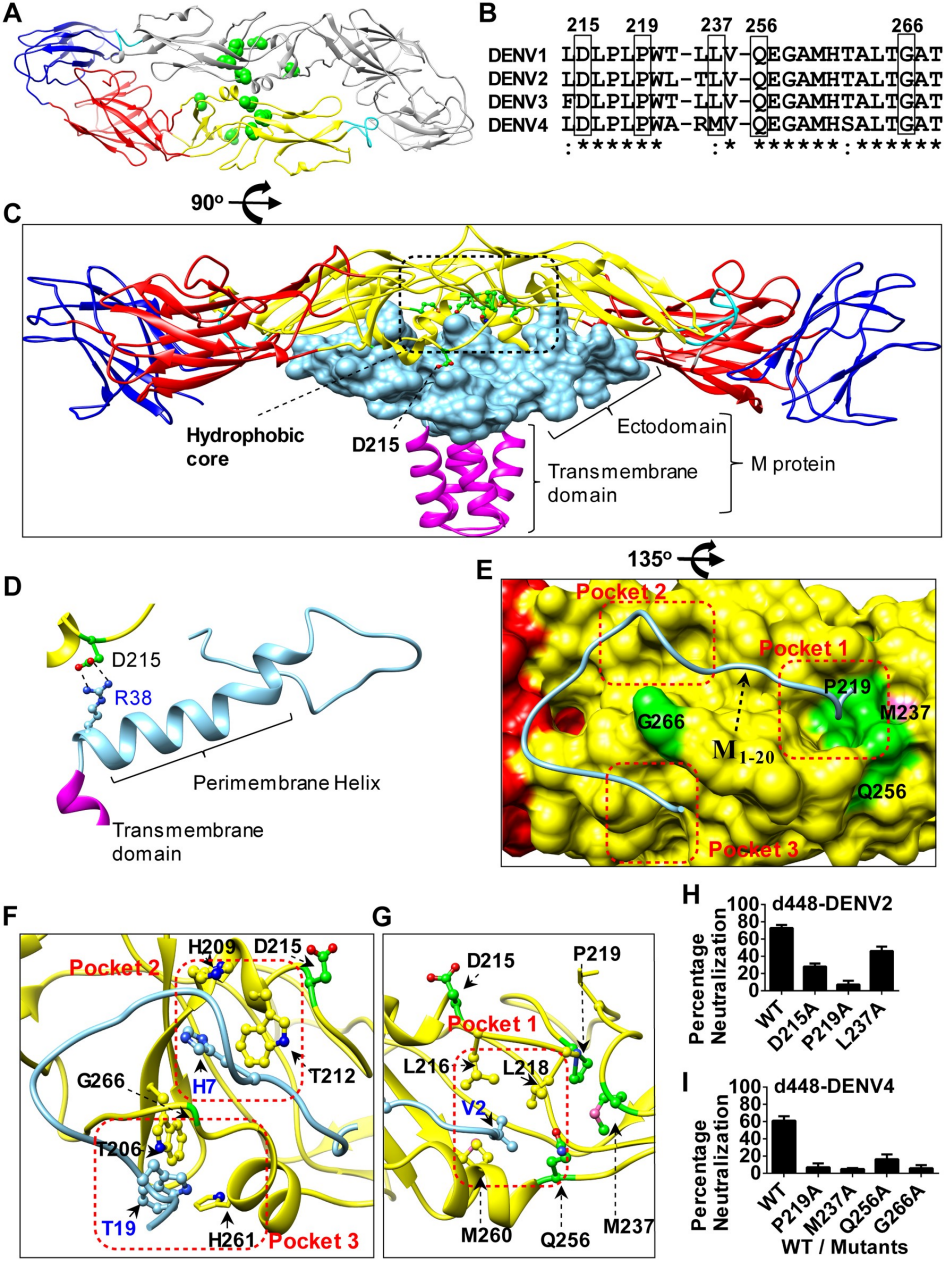
Critical residues for binding of the broadly neutralizing antibody d448. The structural presentation of the critical residues of the epitope on DENV4 domain II. (A) Critical resides (green spheres) are mapped on both monomers of the mature E protein dimer structure (PDB, 1UZG). Domain I is red, domain II yellow, domain III blue and fusion loop cyan. The asymmetric monomer rotated 180 degrees is gray. (B) Sequence alignment of the epitope region on the E protein. Epitope residues D215, P219, Q256, and G266 are conserved among the different serotypes; only one residue M237 in DENV4 is not conserved. (Leucine in the other three serotypes) The NCBI accession numbers of the sequences are ACJ04226 (DENV1), AGS49173 (DENV2), AJA37731 (DENV3) and ACW82884 (DENV4) respectively. (C) Side view of the E:M:M:E heterotetramer. The transmembrane domain of M protein is in magenta, the space-filling model of the ectodomain of M protein is in light blue. Four critical residues (P219, M237, Q256, and G266) of the epitope are located in the hydrophobic core of the E-M interface. One residue (D215), out of the hydrophobic core, contacts the perimembrane helix of the M protein (PDB, 1UZG). (D) Enlarged view shows the contact of D215 with R38 on M protein. The analysis was performed using “Structural Analysis: Distances” of UCSF chimera. (E) Enlargement of the hydrophobic core. The stick model, shown in light blue, presents the first 20 amino acids of M protein (M_1-20_). The space-filling model of E shows the three hydrophobic pockets on E protein, where the M_1-20_ binds. The red dotted lines show the boundaries of the pockets. The critical residues of the epitope are indicated. (F) In pocket 2, H209 and T212 on E form a hydrophobic core with His7 on M, and in pocket 3, T19 on M is encompassed by the recess of T206 and H261 on E. The amino acid G266 forms a barrier between pocket 2 and pocket 3, contacting the conserved residues of H7 and H19 on M protein. (G) The amino acids P219, Q256, M237 are on the edge of pocket 1, consistent with L216, L218, and M260 on E protein, these six residues encompass the valine in the position 2 (V2) on M in the pocket 1 center. Atoms are shown in red for O, blue for N, and magenta for S. Molecular graphic images were produced using the UCSF Chimera (http://www.rbvi.ucsf.edu/chimera). (H) Neutralization percentage of the DENV2 reporter virus particles, and its critical amino acid residue substitution mutants. (I) Neutralization percentage of the DENV4 reporter virus particles, and its critical amino acid residue substitution mutants. Antibody d448 was tested at the concentration of 3 μg/ml. The assays were performed in triplicates and data were analyzed using GraphPad Prism.

Strikingly, antibody d448 showed cross-reactivity to all four serotypes and has an epitope composed of five key amino acids—D215, P219, M237, Q256, and G266 (Green spheres, Fig 5A in DENV4). Those key amino acids are located at the buried interface between the M protein and the ectodomain of the E protein (Fig 5A, 5B and 5C). Four out of the five key amino acids of the antibody d448 epitope are conserved among the four dengue serotypes. DENV4 has a methionine (M) at position 237. The other three serotypes have leucine (L) at position 237 (Fig 5B). A direct interaction occurs between the two charged amino acids, residue D215 on E protein and R38 in the amphipathic perimembrane helix of one M protein (Fig 5D). It has been reported that the M and E proteins contact mainly through three distinct hydrophobic interaction pockets on the E protein [58]. In addition to the key amino acid D215, all the key amino acid residues for mAb d448 binding are in contact with the three hydrophobic pockets (Fig 5E). Hydrophobic pocket 2 is composed of H209 and T212 on E protein. Hydrophobic pocket 3 is composed of T206 and H261 on E protein (Fig 5F). The key amino acid residue of the d448 binding epitope, G266, is located between hydrophobic pocket 2 and pocket 3. G266 creates a barrier for the two hydrophobic pockets, contacting with H7 and T19 on M protein (Fig 5F). Binding of antibody d488 to the epitope directly blocks both pocket 2 and pocket 3 on the E protein. Residues P219, M237, and Q256 in the antibody d488 epitope are adjacent to the hydrophobic pocket 1. This pocket is composed of L216, L218, and M260 on E protein and the V2 on M protein (Fig 5G). Based on the location of those key residues, we propose that antibody d448 interaction with the key structural components in dengue viral coat proteins disturbs the M-E interaction and inhibits virus ‘breathing’ or maturation. To confirm the epitope of d448, we generated the DENV2-RVPs and DENV4-RVPs with Alanine substitutions (D215A, P219A, L/M237A, Q256A, and G266A). However, two DENV2-RVP constructs (Q256A, G266A) and one DENV4-RVP construct (D215A) cannot produce infectious particles with detectable reporter signal, and these were excluded in the neutralization assay (S1 Table). As shown in Fig 5H and 5I, mutations of the residues in the epitope dramatically abolished the neutralizing activity of d448, when compared to the wild type RVP. These results validate the epitope of d448.

### The cross-reactivity of d448 to the flavivirus

**Fig 6:**
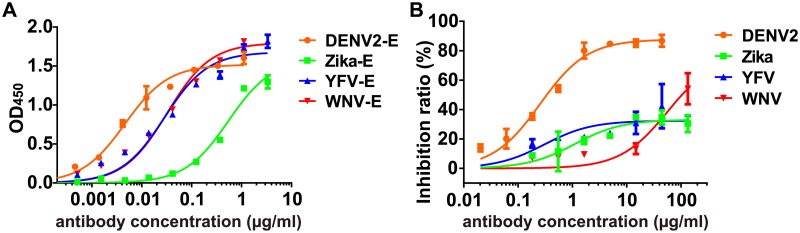
The cross-reactivity of antibody d448 to flaviviruses. (A) Binding of d448 to the E proteins of flaviviruses was analyzed by ELISA. E proteins (100 ng/well) were coated on 96-well plate. Serial dilution of the d448 antibody was added in the coated plate for the detection of the binding, and measurement of the EC_50_. (B) The neutralization activity of d448 was determined by assaying Vero cell infectivity in the presence of serial dilutions of purified antibody. DENV2 (16681 strain) was used as a positive control. Zika virus (SMGC-1 strain), yellow Fever virus (17D strain), and West Nile virus (Kunjin, MRM61C strain) were used in the study.

Interestingly, the interface epitope of d488 is conserved in the flavivirus family (S7 Fig). We hypothesized that d448 might also neutralize other flaviviruses. We first assessed the cross-reactivity of d448 to the purified E proteins of Zika virus, yellow fever virus (YFV), and West Nile virus (WNV). As shown in Fig 6A, d448 binds to YFV E protein with an EC_50_ of 0.028 and WNV E protein with an EC_50_ of 0.032 μg/ml. In contrast, d448 show the weaker binding to Zika E protein with an EC_50_ of 0.5 μg/ml. Next, we evaluated the neutralizing potency of d448 to the four flaviviruses. Antibody d488 exhibited weak neutralizing activity (around 30%) to Zika and WNV at concentrations above 1 μg/ml (Fig 6B). Antibody d488 exhibited minimal neutralizing activity of WNV (about 30% at a concentration of above 30 μg/ml).

### Antibodies cover diverse V-gene sequences

**Fig 7:**
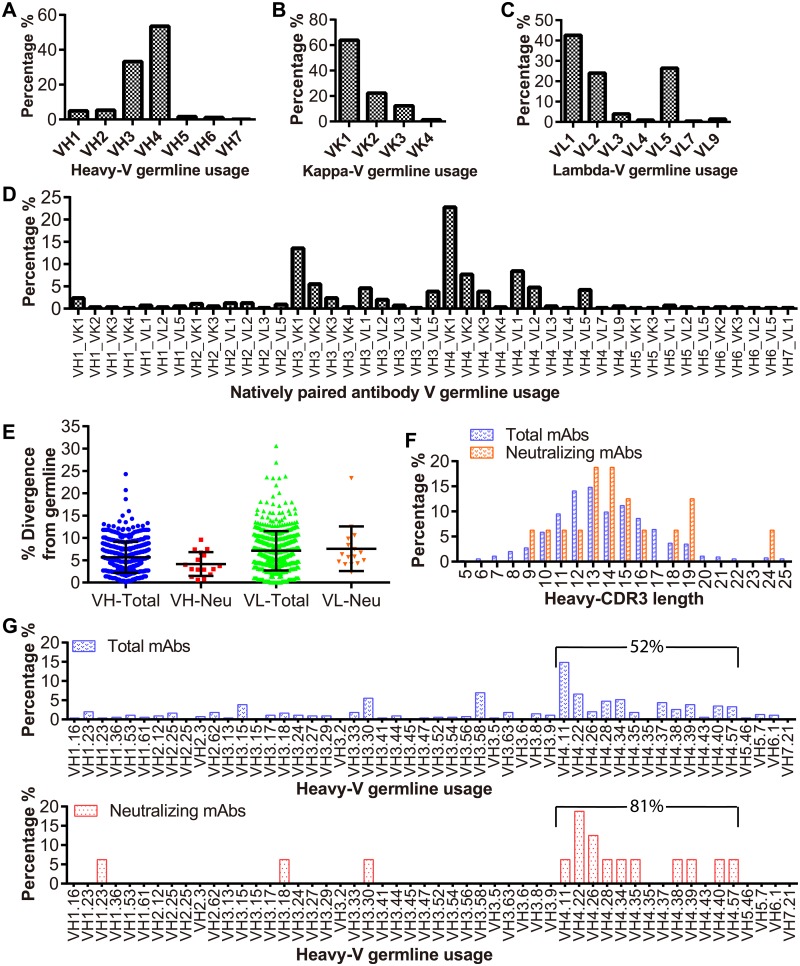
Germline usage and the mutation analysis of the antibodies. General repertoire properties of the rhesus macaque antibodies, including germline gene usage, germline gene divergence and the length of heavy chain complementarity determining region 3 (CDR3). The analysis was performed using IgBLAST with IMGT V domain delineation (https://www.ncbi.nlm.nih.gov/igblast/). The entire panel contains all isolated mAbs and neutralizing mAbs. (A) Heavy chain V-gene germline usage of total mAbs. (B) Kappa chain V-gene germline usage of total mAbs. (C) Lambda chain V-gene germline usage of total mAbs. (D) The natively paired V-gene germline usage of total mAbs. All the heavy chains are associated with their natively paired light chain (kappa or lambda). (E) The germline mutation rate of the native antibodies. Each dot represents one unique sequence. The mean value and the standard deviation are indicated with the black bar, no significant difference regarding the germline gene divergence of the total mAbs and the neutralizing mAbs. The labels are VH-Total: the heavy chain of the total mAbs; VH-Neu: the heavy chain of the neutralizing mAbs; VL-Total: the light chain of the total mAbs; VL-Neu: the light chain of the neutralizing mAbs. (F) The heavy chain CDR3 length of the cloned mAbs (blue), and the neutralizing mAbs (red). (G) The heavy chain V germline distribution of the cloned antibodies (blue), and the neutralizing antibodies (red). The percentage of antibodies with the VH4 germline is shown.

To determine sequence diversity of those antibodies with potent neutralization function, we used NCBI/IgBLAST to analyze the V(D)J-gene family assignment. The V-gene usage for heavy chains of the IgGs shows a high percentage of IgHV3 (36%) and IgHV4 (52%) among the mAbs (Fig 7A). The remaining gene families—IgHV1, 2, 5, 6, and 7—combined to make up only ~9% of the expressed antibody gene repertoires. For the light chain, the Ig-kappa (κ) repertoires predominantly expressed Igκ-V1 (64%), with the remaining gene families accounting for the other 36% (Fig 7B). The Ig-lambda (λ) repertoires predominantly expressed Igλ-V1 (42%). Igλ-V5 accounted for another 25% and Igλ-V2 accounted for 24% (Fig 7C). The profile of V-gene usage for the cloned DENV antibodies is very similar to the distribution of the V-gene families in circulating IgG repertoires of rhesus macaques [59]. To determine the association among heavy chain and light chain gene families, we analyzed gene families for each paired heavy chain and light chain sequence isolated from single antibody-secreting B cells. As shown in Fig 7D, the VH4-VK1 (22%) and the VH3-VK1 (13%) pairs are the top two types of pairing and represent the dominant germline of VH4, VH3, and VK1.

To assess the antibody maturation in DENV neutralizing antibodies in comparison to the entire panel of cloned antibody sequences, we analyzed the rate of antibody V-gene somatic hypermutation (SHM) and the CDR3 length. There were no significant differences in the average mutation rate (Fig 7E). The heavy chain CDR3 length of the neutralizing antibodies is similar to that of the other mAbs, ranking from 5 to 25 amino acids. The majority of mAbs (87%) have a CDR3 length of 9–18 amino acids in both groups (Fig 7F). However, it is worth noting that the cross-reactive neutralizing antibodies, d462 and d448, each have a long heavy chain CDR3 (19 amino acids). The whole panel of 780 antibodies has an average of 14 amino acids in the heavy chain CDR3. We further analyzed the VH germline of the neutralizing antibodies in comparison with the usage of total cloned antibodies. Distribution of the VH4 germline of neutralizing antibodies was 81%, while the antibodies overall showed 52% usage of VH4 (Fig 7G). Interestingly, the six potent neutralizing antibodies express the VH4 germline (S2 Table). In contrast, the germlines for light chain sequences did not show any preferential distribution (S8 Fig, S2 Table).

## Discussion

The objective of this study was to: (1) evaluate the efficacy of an experimental DENV vaccine by the elicited neutralizing antibodies, and (2) characterize the epitopes recognized by the mAbs and examine their potency of neutralization. Although NHPs typically do not develop the disease as humans do following dengue viral infection, they are still the most valuable models available for evaluating the efficacy of experimental dengue vaccines [16,55]. In our previous study, the primer-boost strategy was shown to induce balanced type-specific and broadly neutralizing humoral responses to the desired DENV serotypes in rhesus macaques [20,21]. Therefore, characterization of macaque mAbs against dengue vaccine is valuable for better understanding the immunologic responses of NHPs. The NHP antibodies with epitopes more closely related to those of human antibodies may better inform the preclinical development of vaccines. They are particularly relevant if antibodies can be characterized that recognize diverse epitopes that are neutralizing antibody binding sites also targeted by the human antibody repertoire.

In this study, we cloned and analyzed a large panel of DENV reactive antibodies from single ASCs including both serotype specific and broadly neutralizing mAbs. Among the 780 antibodies cloned, 130 showed binding to the DENV-80E protein, with a positive rate of 17% in ELISA. We used LAV displaying dimeric E protein as a priming vaccine and monomer subunit E proteins to boost the immune response. With the booster dose, only about half of the E-antibodies binding to monomeric E protein could be enriched, according to the previous human study [51]. Nevertheless, we were able to isolate six potent antibodies with diverse neutralizing epitopes from the rhesus macaque model. These included antibodies binding to the lateral ridge of DIII (d182, d511, d628), the I-III hinge (d182), the bc loop adjacent with the fusion loop (d559) of DII, the β-strands and the loops of DI (d462), and the interface of E protein with the M protein in DII (d448). Epitopes within some of those motifs or domains have been reported for dengue neutralizing antibodies from different species including mouse, chimpanzee, and humans [46,50,54]. As expected, the vaccination using monomeric E protein in our study did not yield any neutralizing antibodies targeting E dimer epitopes as have been reported for antibodies from human patients [51,60]. Nevertheless, isolation of potent neutralizing antibodies with diverse epitopes in this study provides a strong validation for the efficacy of the vaccine and immunization strategy.

DIII was identified as the dominant epitope for the mouse antibodies but not for human antibodies [42]. Mouse antibodies elicited by DENV1-4 immunization target DIII and exhibit potent neutralization [38,61–63]. Epitope mapping of these antibodies, with random recombinant DIII mutants, identified the lateral ridge in DIII as the target of strongly neutralizing antibodies. Similarly, we identified three potent neutralizing antibodies recognizing critical residues T/D329 (d182, d511, d628) and K361 (d511, d628) in the lateral ridge of DIII. We propose that the lateral ridge epitope represents an important neutralizing site on DENV for rhesus macaque. In this study, we also isolated neutralizing antibodies recognizing the DI-DIII hinge (d182), DI (d462), and DII (d559, d448). Prior to our study, little was known as to how rhesus macaque neutralizing antibodies recognized DENV. A non-DIII antibody response has been shown to be induced by live DENV infection in rhesus macaque. However, in the same study, the alphavirus vector-based dengue E dimer vaccine-induced predominantly DIII neutralizing antibodies in rhesus macaques [64]. According to our data, the existence of other epitopes in distinct regions should not be ruled out for rhesus macaque antibodies. The deviations of the epitopes from those reported in different studies may be due to various factors that cause differences in the B cell repertoire. Namely, variations in the immunization strategy, primary screening, and genetic profiles of the rhesus macaques could be reflected in the antibody response.

Identifying the target epitopes of the antibodies, generated by rhesus macaque post vaccination, is the best way to understand the molecular determinants of the NHP immune response to DENV. It is worth noting that the epitopes we reported in this study are critical for human dengue antibodies as well as for dengue antibodies from other species [46,54,65]. The mAb d182 recognizes V300 in the I-III linker (E299-304) and the T329 in DIII. A panel of mouse DIII-neutralizing antibodies also targets this region [38]. The I-III linker is essential for dengue virus particle assembly in the cell and interaction of E protein with the heparin receptor [57]. These results suggest that d182 may inhibit virus entry by blocking DENV binding to the cell surface heparin sulfate, which is the initial step of infection. We identified that the mAb d559 binds the bc loop of DII, adjacent to the fusion loop. Previously, the potent human antibody 1C19 was reported to recognize this epitope and could neutralize all four serotypes better than any antibodies targeting the fusion loop [46]. Our study also indicates that d462 recognizes DI epitopes are overlapping that targeted by chimpanzee neutralizing antibody 5H2 [50]. This result shows that the d462 may block the virus infection by the same mechanism, that is, by preventing conformational change during fusion. The unique epitopes revealed by this study suggests a focus for rational vaccine design based on the novel immunogens of DENV.

This study, for the first time, identified a broadly neutralizing DENV mAb d448 with a novel epitope at the interface of the E and M proteins. The mAb d448 is likely binding to the E protein in the immature DENV virus, interfering with DENV “breathing” by blocking the E-M protein interaction [66,67]. As the E protein dynamically switches between dimers and trimers during DENV “breathing,” mAb d448 may block the E protein transition from dimer to trimer.

The dengue E protein bears greater than 50% homology to the Zika virus E protein [68]. Monoclonal antibodies isolated from dengue infected patients could inhibit Zika infection [69,70]. However, the broadly dengue neutralizing antibody d448 identified in this stuy showed weak neutralizing activity to Zika and other flaviviruses. It has been reported that “group B” antibodies isolated from infected human patients also interact with the D215 and A267 residues in the DII domain in DENV4, which partially overlaps with the epitope of d448 [71]. The “group B” antibodies are relatively weaker neutralizers *in vitro*, which may explain the fact that antibody d448 is not a particularly strong neutralizer when compared with other neutralizing antibodies isolated in this study. Significance of the d448 epitope in dengue neutralization in the clinical setting warrant further study.

The cross-reactivity of serum from patients with DENV and Zika virus, and the antibody dependent enhancement (ADE) of infection has presented a challenge for accurate identification of the infecting agent, and diseases control [72,73]. This raises questions regarding the role of the DENV antibodies in protective immunity and impact on pathogenesis. In our study, most of the antibodies are relatively weak in neutralizing activity or binding affinity. The less potent cross-reactive antibodies can induce ADE to different dengue serotypes and the Zika virus. We have not yet assessed the ADE effect of the isolated rhesus antibodies. Further studies to determine whether the rhesus antibodies can induce ADE activity will be essential for understanding the efficacy of the experimental dengue vaccine. The NHP could be used as a model for the study of ADE in flavivirus pathogenesis [74].

In our study, two neutralizing antibodies (d511, d628) recognizing the lateral ridge of DENV2 DIII were isolated from two different macaque subjects. They share similar CDR3 sequences and both heavy and light chains are of the same germlines. The “unique” paired genetic restriction to specific V-genes, and CDR3 sequences indicate that the antibodies might be dominant in the DENV2-neutralizing lineage of the B cell repertoire. Similarly, detection of the dominant VCR01 classes of antibodies has been reported in HIV studies and the restricted IGHV1-69 germline in influenza studies [75,76]. Based on the analysis of our entire panel of 780 mAbs, a similar divergence in antibody germlines was shown in this study as those reported for normal and HIV-infected macaque [59,77]. However, neutralizing antibodies are biased towards the VH4 germline, and the light chain did not show a clear preference for any particular germline. These results indicate that the heavy chain may be subject to more selection pressure on humoral response to this vaccination.

In summary, this study profiled antibody responses to an experimental vaccine in NHP and laid a foundation for further evaluation of the vaccine in the preclinical trial. The novel neutralizing antibodies can be evaluated for therapeutic potential for the treatment of dengue infection. Epitopes of the very potent neutralizing monoclonal antibodies offer insights for better dengue vaccine design.

## Materials and methods

### Immunization and single cell sorting of rhesus macaque antibody-secreting cells

The live attenuated vaccine (gifted by Dr. Stephen S. Whitehead, National Institutes of Health) comprised dengue types 1–4 (rDEN1-rDEN1Δ30–1545; rDEN2-rDEN2/4 Δ30(ME)-1495,7163; rDEN3-rDEN3Δ30/31-7164; and rDEN4-rDEN4 Δ30–7132,7163,8308). The tetravalent dengue subunit vaccine (V180, Merck and Co. Inc.) comprises truncated forms of envelope proteins (DEN-80E), derived from strains of all four dengue virus serotypes (DENV1 strain 258848, DENV2 strain PR159 S1, DENV3 strain CH53489, and DENV4 strain H241). The immunization strategy has been reported previously [22]. Briefly, all the subjects received the live attenuated vaccine subcutaneously at 0 weeks and then received subunit DEN-80E vaccine formulated with Alhydrogel adjuvant (Brenntag Biosector) intramuscularly at 16 weeks. Bleeds were taken 7 days after a boost. PBMCs were isolated and stained for FACS sorting as described [22]. The targeted sorting population was CD3^−^/CD19^low to +^/CD20^– to low^/ sIgG^−^/CD38^+^ /CD27^– or +^. The sorted cells were stored at -80°C until analysis.

### Ethics statement

Indian Rhesus macaques were domestically bred, raised and maintained at New Iberia Research Center (NIRC) of University of Louisiana at Lafayette, New Iberia, LA, USA. Flavivirus naïve (i.e. sera negative for Dengue virus 1,2,3,4 and West Nile virus) monkeys of either sex, weighing more than 3 kg were used in this study. The animal studies were approved by the University of Louisiana at Lafayette Institutional Animal Care and Use Committee (IACUC) and conducted in accordance with the US Public Health Service (PHS) Policy on Humane Care and Use of Laboratory Animals. All animals were socially housed for the study. The dimensions of the cage for paired housing is 8.6 (floor area, square footage) by 30 (height, inch). Each animal’s holding cage was cleaned daily. Animals were provided with object(s) to manipulate or explore. Harlan Teklad Monkey Chow, or its equivalent, was provided daily in amounts appropriate for the size of the animal. The basic diet was supplemented with fruit and novel treats including small quantities of fresh fruits, nuts, or seeds, 2 to 3 times weekly as part of the site’s environmental enrichment program. Tap water was provided ad libitum via automatic watering device. No contaminants are known to be present in the food or water which would interfere with the results of this study. Food was withheld at least 2–3 hours on days of study procedures to insure safe sedation and was offered upon recovery from sedation. Animals were observed twice daily throughout the study for any abnormal clinical signs, signs of illness or distress. All animals were returned to the colony at NIRC at the end of the study.

### Cloning antibody sequences and antibody expression

The natively paired antibody genes were cloned from single cells, as previously described [22]. Briefly, reverse transcription was carried out to synthesize the cDNA from each single cell using a one-step cDNA synthesis kit (Bio-Rad) according to the manufacturer’s protocol. Antibody variable regions were amplified by a two-step nested PCR with 3.5 μl of the template cDNA. All PCR reactions were performed in 96-well plates in a volume of 25 μl per well with our validated primers [22]. The purified gene fragments were inserted into the vector with the human IgG1 constant region. The human-macaque chimeric antibodies were expressed in HEK293-F cell lines (Thermo Fisher Scientific). IgG sequences were analyzed for their CDR3 length, germline IgH, Igκ, and Igλ V-gene family distribution using IgBLAST with IMGT V domain delineation (https://www.ncbi.nlm.nih.gov/igblast/). The method of antibody expression in mammalian cells and purification by Protein A has been described previously [78,79]. Briefly, 0.5 μg aliquots of heavy chain plasmid and light chain plasmids (total of 1 μg plasmids/1 ml of the transfected cell) were co-transfected into HEK293-F cell lines for transient expression with TrueFect reagent (United BioSystems). The supernatants were harvested 7 days after transfection. Antibodies were purified with Protein A resin (Repligen) according to the manufacturer’s instructions.

### Antibody binding ELISA

Recombinant flavivirus envelope protein was coated on the 96-well plates with the concentration of 1 μg/ml at 4°C overnight. Plates were blocked with 3% BSA in PBS, then incubated with the supernatants or purified mAb in 3-fold serial dilutions. After incubation for 1.5 hours at room temperature, the plates were washed 3 times with PBST (0.5% Tween-20 in PBS), followed by addition of horseradish peroxidase (HRP) coupled goat anti-human IgG (Sigma) and detected using TMB Substrate (Thermo Fisher Scientific) for absorbance signal at OD_450_ nm using a 96-well plate reader (Molecular Devices). Samples having an OD_450_ nm of above or equal to 0.1 were designated as positive binders. The OD_450_ of native control were below 0.4. All assays were replicated three times.

### Dengue microneutralization assay

Micro-neutralization assay was based on staining viral E protein with near-infrared fluorescent dye (IRD)-labeled reagents, as previously described [80]. Antibody concentration titrations were used in each assay and concentration to generate 50% neutralization activities (NT_50_) was derived from the antibody titration curve using GraphPad Prism (GraphPad Software Inc.) with a nonlinear regression and 4-parameter curve fitting model.

For the rhesus macaque sera tittering, two-fold serial dilutions of heat-inactivated serum samples from vaccinated animals were incubated for 1 h at 37°C with 50 PFU of each DENV. This virus-serum mixture was then added onto Vero cells in 96-well plates and incubated for 4 days. Serum end-point neutralization titers (LiCor_50_) were defined as the reciprocal of the highest serum dilution that blocks 50% of the DENV infection when compared to virus control included on each assay plate, using an infrared Odyssey Sa imaging system (Li-Cor Biosciences) [81].

### Neutralization assay by flow cytometry

Zika (SMGC-1 strain), yellow Fever virus (17D strain), and West Nile virus (Kunjin, MRM61C strain) were propagated in Vero cells as previously described [82,83]. For antibody neutralizing activity of d448, the flow cytometry-based neutralization assay was conducted using our previous method [82,83]. Briefly, 2×10^5^ of Vero cells were seeded in each well of 24-well plate 24 h before virus infection. The purified antibody d448 was serially diluted and incubated with the virus (5×10^3^ PFU) for 1 h at 37°C. Vero cells were then incubated with the antibody and virus mixture for 40 h at 37°C supplied with 5% CO_2_. Afterward, infected cells were collected by trypsin digestion, fixed and permeabilized by Fixation and Permeabilization solution (BD Biosciences); and stained with pan-flavivirus antibody Z6 (for ZIKV, YFN, and WNV) at 2 μg/ml [82]. Cells were stained with FITC-conjugated secondary antibody on ice for 30 m. The percentage of positive cells were measured using BD FACSCanto II. Sigmoidal neutralization curves were generated using GraphPad Prism 5.

### Determination of antibody binding kinetics using surface plasmon resonance (SPR) method

The CM5 sensor chip was pre-activated according to the manufacturer’s instructions (Biacore T100, GE Healthcare). The dengue antigens (DEN-80E) were immobilized on the sensor chips and reached targeted 1500 RU. Each antibody (analyte) was serially diluted 2 folds down with concentration ranges at 0–200 nM in running buffer. Association and dissociation were conducted at a flow rate of 30 μl/min with a 5-min association followed by 5-min of dissociation, at 25 °C. Then, the chip was regenerated by two 20-second pulses of 3 M MgCl_2_. The data was analyzed using the standard Biacore T100 evaluation software with the 1:1 Langmuir binding model for *ka*, *kd* and *K*_*D*_ determination.

### Antibody binning

An Octet equipped with protein A-coated sensor (ForteBio, Octet RED96) was used to bin DEN4-80E with the mAbs d462 and 5H2. First, 30 μg/ml of d462 and 5H2 were captured onto protein A sensor for 600 seconds. Followed by a blocking antibody (200 μg/ml) to saturate the unoccupied sensors for 300 seconds, the DEN4-80E was loaded in an association step onto the sensor at 30 μg/ml for 300 seconds. The Sensors with DEN4-80E provide a surface for the binding of the secondary mAbs d462, 5H2, d559, d182. If the second antibody showed mass accumulation to the sensors, it was considered to bind to a different epitope than the captured antibody. The mAb d559 was used as positive control, d182 as a negative control.

### Epitope mapping of monoclonal antibodies

Epitope mapping was conducted using comprehensive mutation libraries made of prM/E from all four DENV serotypes [53]. The DENV1 library consists of random mutations introduced at each residue of the DENV1 prM/E polyprotein (strain WestPac), while for DENV2 (strain 16681), DENV3 (strain CH53489), and DENV4 (strain 341750), each prM/E residue was individually changed to alanine (and alanine residues to serine). All mutant clones were sequence confirmed and arrayed into 384-well plates (one mutation per well). Mutagenesis achieved >97% coverage of prM/E residues for each serotype, a total of over 2,400 mutations. Antibody d182 was mapped on the DENV1 library, antibodies d511 and d628 on the DENV2 library, and antibodies d462, d448, and d559 on the DENV4 library. For each screening, a DENV prM/E library was expressed in HEK-293T cells and assayed by immunofluorescence for mAb binding as described previously [84]. In some cases, mAbs were also screened as Fabs after conversion by papain digestion. Antibodies were detected using AlexaFluor488-conjugated secondary antibody (Jackson ImmunoResearch Laboratories). Cells were washed three times with PBS/+0.2% saponin followed by two washes in PBS. Mean cellular fluorescence was detected using a high-throughput flow cytometer (HTFC, Intellicyt). Antibody reactivity against each mutant protein clone was calculated relative to wild-type protein reactivity by subtracting the signal from mock-transfected controls and normalizing to the signal from wild-type prM/E-transfected controls. Mutations within clones were identified as critical to the mAb epitope if they did not support reactivity of the test mAb but supported reactivity of other antibodies. This counter-screen strategy facilitates the exclusion of DENV protein mutants that are misfolded or have an expression defect. The mAbs D004, D449, D168, D413, D341, and D195 were used as positive control.

### Neutralization assays with DENV reporter virus particles

DENV-RVPs were produced in HEK-293T cells by co-transfection with plasmids of DENV structural genes (CprME) and WNV subgenomic replicon. The plasmids encoding DENV2-CprME (strain: 16681) and the WNV subgenomic replicon with Renilla luciferase reporter gene (pWNII-rep-Ren-IB) were described previsuly [85]. We replaced the DENV2-CprME with CprMEs from DENV1 (strain: Hawaii) and DENV4 (strain: H241), respectively, to generate other serotype RVPs. The amino acid residue substitution mutants were created using site directed mutagenesis by PCR. Briefly, HEK-293T cells were plated at a density of 400,000 cells per well in 12 well plate overnight, the cells were then co-transfected with 1.5 μg of DENV structural gene and 0.5 μg of WNV subgenomic replicon. After 4 hours, the culture medium was replaced with low glucose formulation of DMEM (7% FBS), then the cells were cultured at 33°C under 5% CO2 for another 48 hours. The RVPs in the supernatants were harvested by passing through 0.45 μm filters. For infection assay in 96-well plate, Vero cells were added to each well at a density of 30,000 cells per well in 100 μl of DMEM complete medium (7% FBS), then 100 μl RVP were added to each well with the neutralizing antibodies at the final concentration of 3 μg/ml followed by incubation at 37°C for 48 hours. The infected cells were lysed in 20 μl of lysis reagent and assayed using luciferin-containing substrate (Promega: Renilla Luciferase Assay System, E2810). Luminescence was measured using a luminometer. The relative titer of the mutants was normalized to the wildtype RVP. The assays were performed in triplicates and data were analyzed using GraphPad Prism.

### Sequence alignment and display of E protein epitope

Protein sequence alignment of DEN-80E of DENV1, DENV2, DENV3, and DENV4 was generated and displayed using ClustalW2 and ESPript 3.x [86,87]. The NCBI accession numbers are ACJ04226 (DENV1), AGS49173 (DENV2), AJA37731 (DENV3) and ACW82884 (DENV4), respectively. The E protein model and the amino acid representation of the epitopes were displayed using UCSF chimera (http://www.rbvi.ucsf.edu/chimera). The protein data bank (PDB) accession numbers of the dengue envelope protein are 1UZG [88], 3G7T [89], 1OK8 [25], 3UC0 [50], 3C6E [24], 3J27 [67].

## Supporting information

S1 Fig Flowchart of the antibody isolation and cloning process. Single antibody-secreting B cells were isolated from rhesus macaque immunized with the dengue vaccine as reported previously. Two vaccine candidates, tetravalent dengue live attenuated virus vaccine, and tetravalent recombinant dengue subunit vaccine (DEN-80E) was administered using different regimens. All animals received the live attenuated vaccine subcutaneously (SC) at 0 weeks and then received subunit DEN-80E vaccine intramuscularly (IM) at 16 weeks. The peripheral blood mononuclear cells (PBMCs) were isolated after 7 days of boost. Antibody-secreting cells were sorted as single cells into individual wells of a 96-well plate. Antibody variable region genes were amplified and cloned into the antibody expression vector of human IgH, Igκ, and Igλ respectively. The heavy-chain and light-chain expression vectors were co-transfected into HEK293-F cells for transient expression in 12-well plate (1.5 ml). The binding ELISA was performed with the supernatant antibodies for the initial binding screen. The positive binding antibodies were then expressed in large-scale volume (20 ml), and purified by protein A chromatography. The characterization of the antibodies was confirmed by binding titration to the antigen and neutralizing assay to the dengue virus (DENV).
(TIF)

S2 Fig Analysis of binding properties of purified antibodies. The DEN-80E is coated on the plate for ELISA binding titration. The purified antibodies were diluted, and incubated with the coated antigen. The binding of the antibodies were detected by OD_450_ with duplication. The curves were fitted by the one-site specific binding model.
(TIF)

S3 Fig SPR Sensorgrams used to determine kinetic constants of neutralizing mAbs. Surface plasmon resonance sensorgrams show the antibody binding to the immobilized DEN-80E (DEN1-80E, DEN2-80E, DEN3-80E, and DEN4-80E). The y-axis shows the binding of resonance units (RU), and the x-axis shows the elapsed time (second, s). The antibodies were serially diluted 2-fold down with concentrations of 200–6.25 nM. The kinetic analysis was performed at a panel of different concentration of the antibodies with a contact time of 300s followed by 300s dissociation. All measurements were performed at least three times. Data were analyzed by using BIA evaluation software 4.1, and fitted to 1:1 Langmuir binding model. The antibodies are d182, d448, d462, d511, d559, d622, and d628 as indicated.
(TIF)

S4 Fig Epitope mapping of the potent neutralizing antibodies. The ‘Shotgun Mutagenesis’ epitope mapping was performed by subjecting expression constructs for prM/E from all four DENV serotypes to high-throughput mutagenesis to generate comprehensive mutation libraries. The library consists of random mutations (to alanine, and analnie to serine) introduced at each residue of the prM/E polyprotein, while for DENV1 (strain WestPac), DENV2 (strain 16681), DENV3 (strain CH53489), and DENV4 (strain 341750). For screening, the DENV prM/E library was expressed in HEK-293T cells and assayed by immunofluorescence for the testing mAb binding to each clone. The antibody reactivity against each mutant protein clone was calculated relative to wild-type protein reactivity by subtracting the signal from mock-transfected controls (% Wildtype Reactivity). The mAb d182 (A) was mapped on the DENV1 library, d511 (D), d559 (E) and d628 (F) on the DENV2 library, and d462 (C), d448 (B), on the DENV4 library. The positive binding antibodies are D004, D449, D168, D413, D341, and D195 as indicated. Error bars represent the average of three measurements ± SD.
(TIF)

S5 Fig Multiple sequence alignment of the Envelope protein. The envelope protein sequence alignment of DEN-80E of DENV1, DENV2, DENV3, and DENV4 was generated and analyzed using ClustalW2 (http://www.ebi.ac.uk/Tools/msa/clustalw2/) and ESPript 3.x (http://espript.ibcp.fr/ESPript/ESPript/). The NCBI accession numbers are ACJ04226 (DENV1), AGS49173 (DENV2), AJA37731 (DENV3) and ACW82884 (DENV4) respectively. Identical residues are shown as white text on a red background, and similar residues are shown as red text. The secondary structure of DEN-80E (PDB: 1OK8) are displayed above the residue numbers. The β-strands are labeled with different letters, as A_0_ to I_0_ for the domain I, a to l for domain II, and A to G for domain III. The residues on the epitope are marked with the indicated antibody under the alignment, respectively.
(TIF)

S6 Fig The macaque mAb d462 and chimpanzee mAb 5H2 bind to overlapped epitope on DEN4-80E. The real-time competitive binding of d462 and 5H2 was determined using an epitope binning format on Octet RED96 biosensor. The mAb d462 (A) or 5H2 (B) was first captured using protein A biosensor (step 1), followed by a blocking antibody to saturate the unoccupied sensors (step 2). The DEN4-80E was incubated to bind to the captured antibody (step 3). Finally, a secondary antibody was allowed to bind to the DEN4-80 on the sensors (step4). The binding of the secondary antibody will be blocked if it binds to the overlapped epitope with the captured antibody. An isotype control monoclonal antibody d182 was used as negative control (green line). The DENV4 specific mAb d559 was used as positive control (brown line). The d462 (blue line), 5H2 (red line) bind to DEN4-80E competitively.
(TIF)

S7 Fig Protein sequence alignment of Envelope proteins of the flaviviruses. E proteins of Dengue 1 (DENV1), DENV2, DENV3, DENV4, ZIKA virus, Japanese encephalitis virus (JE), West Nile virus (WNV), and the yellow fever virus were aligned. Amino acids in red are those conserved among the flavivirus family. Residues highlighted in yellow are those on the epitope of the antibody d448. The GeneBank accession number of the sequences are: ACJ04226 (DENV1), AGS49173 (DENV2), AJA37731 (DENV3), ACW82884 (DENV4), ARK18853 (ZIKA), AFO42844 (JE), ACI95758 (WNV), and AAA92702 (yellow fever virus).
(TIF)

S8 Fig Germline usages of antibody light chains. General light chain germline usage of the rhesus macaque antibodies. The analysis was performed using IgBLAST with IMGT V domain delineation (https://www.ncbi.nlm.nih.gov/igblast/). The entire panel contains all isolated mAbs and neutralizing mAbs. **(A)** The kappa chain V germline distribution of the cloned antibodies (blue), the neutralizing antibodies (red). **(B)** The lambda chain V germline distribution of the cloned antibodies (blue), the neutralizing antibodies (red).
(TIF)

S1 Table The relative titer of the reporter virus particles with residue substitution. (DOCX)

S2 Table Germline usage and the CDR3 sequence of the potent neutralizing antibodies. (DOCX)
