# Supplementary figures and images for: Potent neutralizing antibodies elicited by dengue vaccine in rhesus macaque target diverse epitopes
Source: PLoS Pathog. 2019 Jun 6;15(6):e1007716. doi: 10.1371/journal.ppat.1007716 (PMC6553876; doi:10.1371/journal.ppat.1007716)

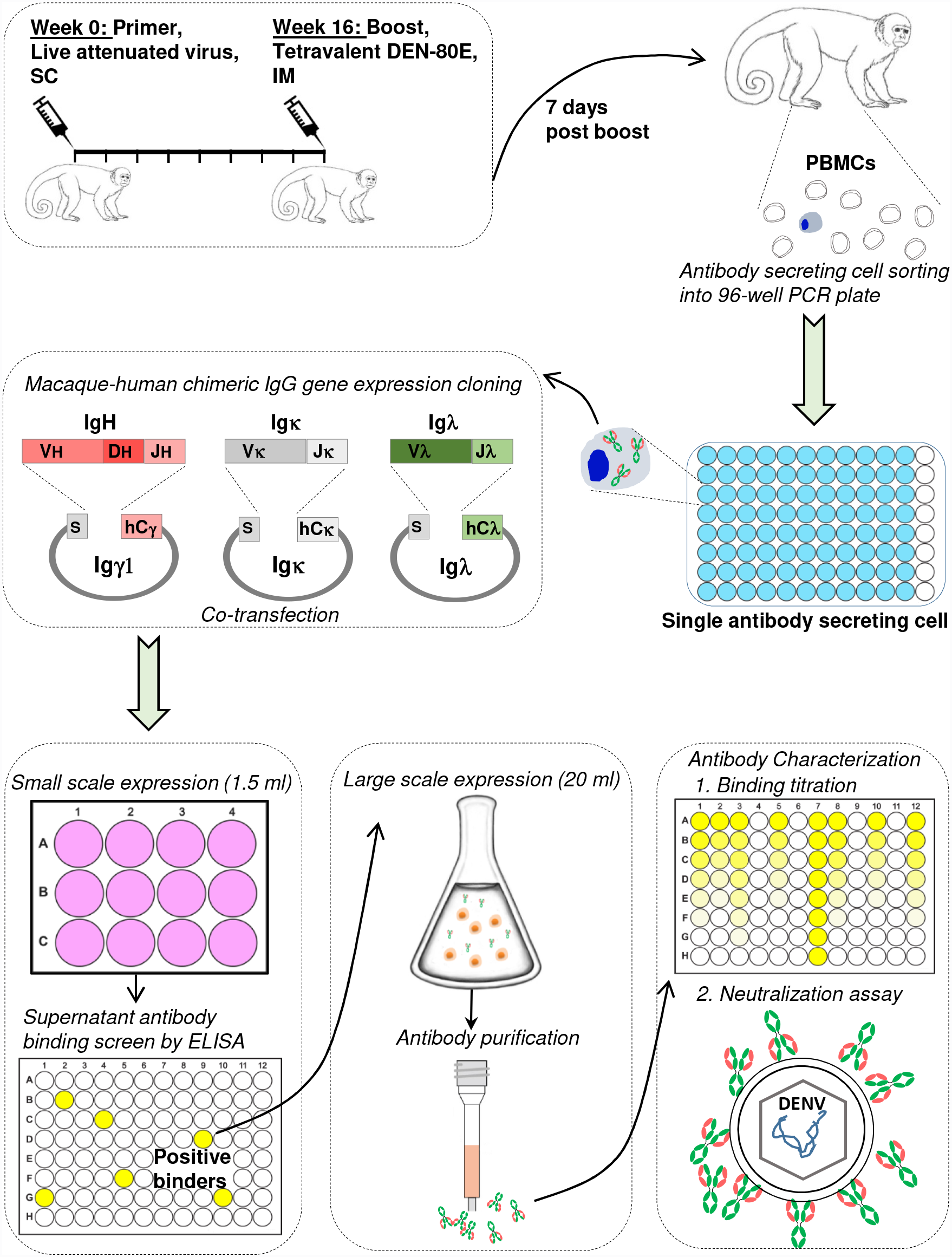

Supplement: S1 Fig — Single antibody-secreting B cells were isolated from rhesus macaque immunized with the dengue vaccine as reported previously. Two vaccine candidates, tetravalent dengue live attenuated virus vaccine, and tetravalent recombinant dengue subunit vaccine (DEN-80E) was administered using different regimens. All animals received the live attenuated vaccine subcutaneously (SC) at 0 weeks and then received subunit DEN-80E vaccine intramuscularly (IM) at 16 weeks. The peripheral blood mononuclear cells (PBMCs) were isolated after 7 days of boost. Antibody-secreting cells were sorted as single cells into individual wells of a 96-well plate. Antibody variable region genes were amplified and cloned into the antibody expression vector of human IgH, Igκ, and Igλ respectively. The heavy-chain and light-chain expression vectors were co-transfected into HEK293-F cells for transient expression in 12-well plate (1.5 ml). The binding ELISA was performed with the supernatant antibodies for the initial binding screen. The positive binding antibodies were then expressed in large-scale volume (20 ml), and purified by protein A chromatography. The characterization of the antibodies was confirmed by binding titration to the antigen and neutralizing assay to the dengue virus (DENV). (TIF) [file ppat.1007716.s001.tif]

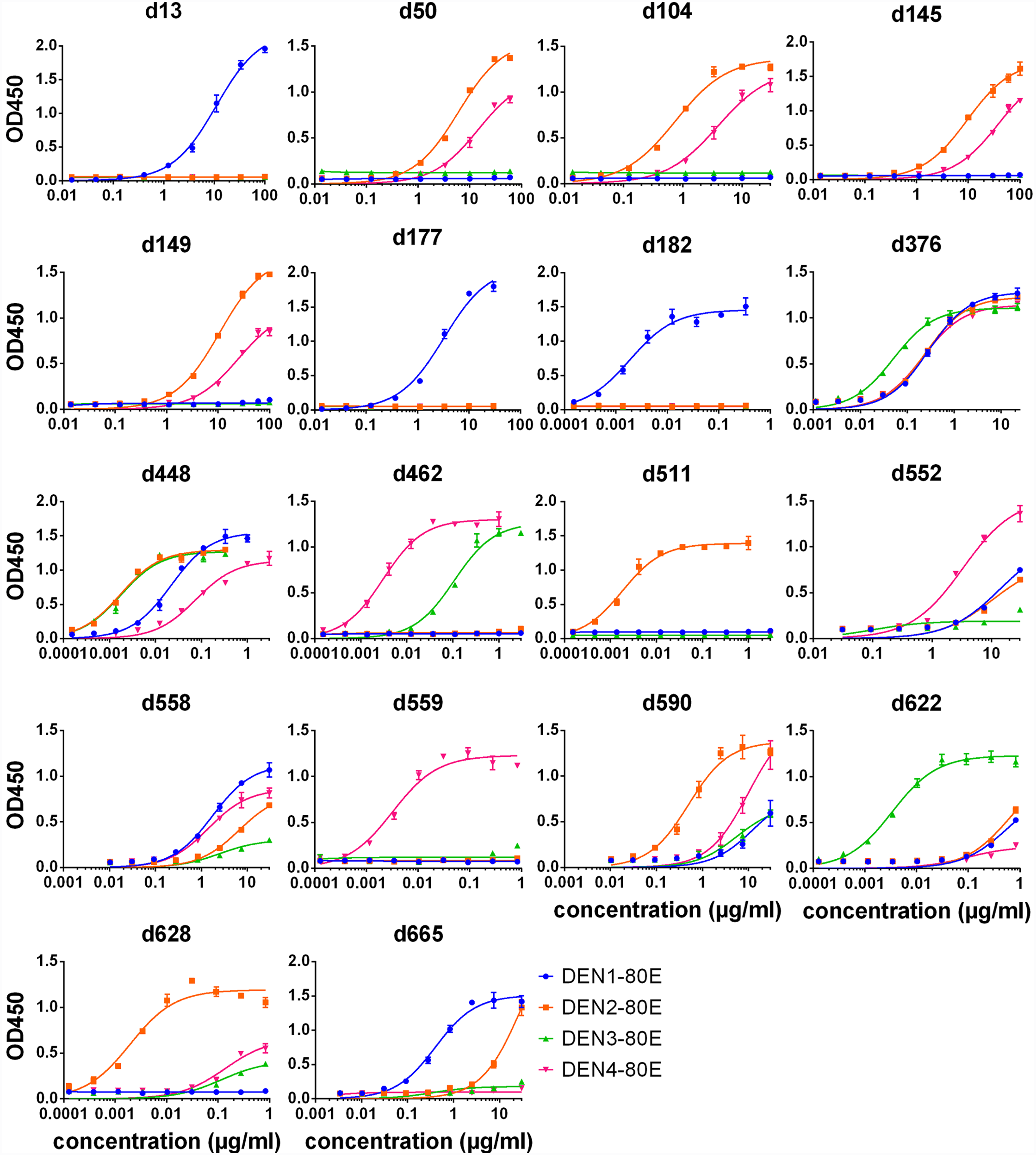

Supplement: S2 Fig — The DEN-80E is coated on the plate for ELISA binding titration. The purified antibodies were diluted, and incubated with the coated antigen. The binding of the antibodies were detected by OD450 with duplication. The curves were fitted by the one-site specific binding model. (TIF) [file ppat.1007716.s002.tif]

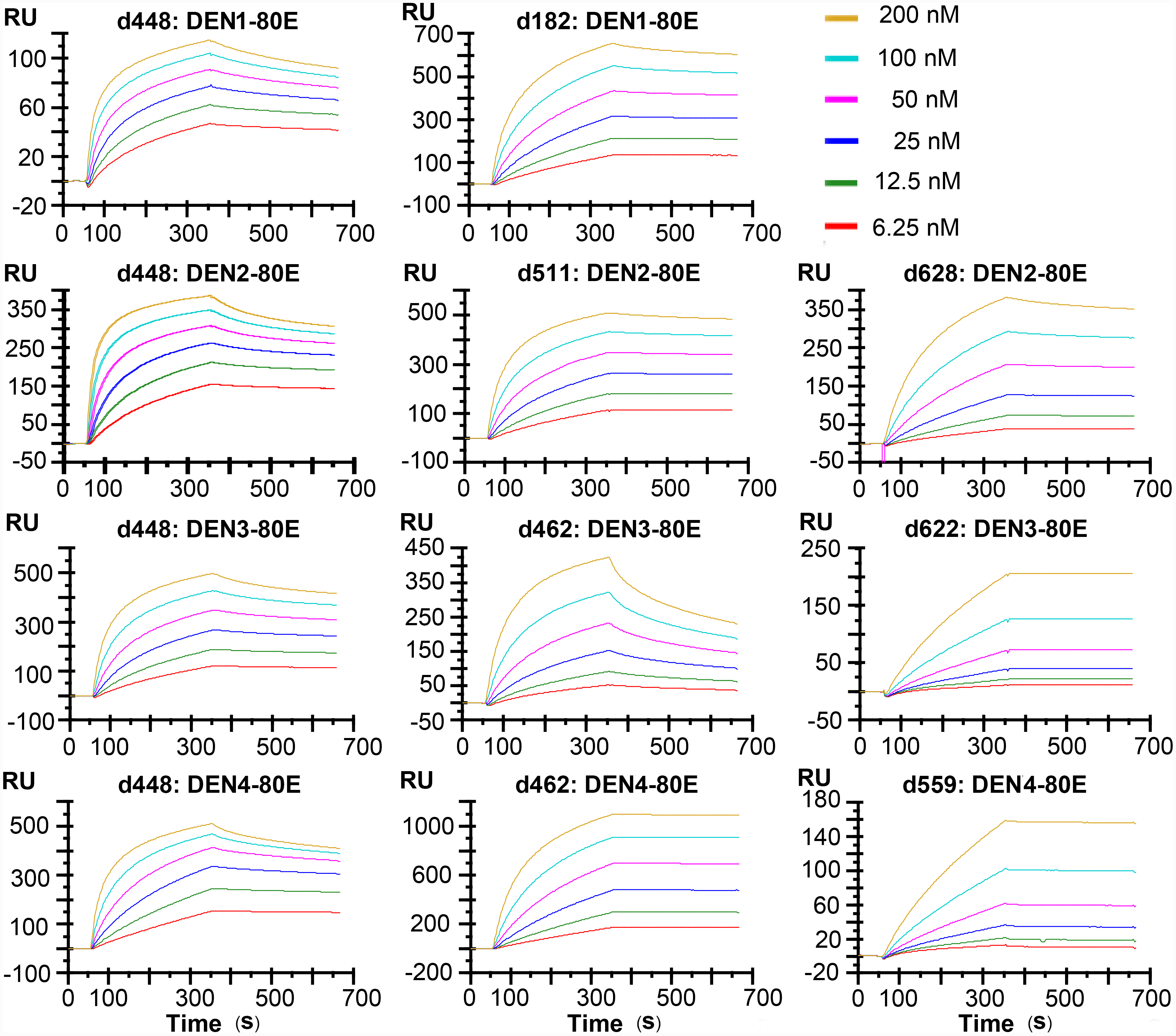

Supplement: S3 Fig — Surface plasmon resonance sensorgrams show the antibody binding to the immobilized DEN-80E (DEN1-80E, DEN2-80E, DEN3-80E, and DEN4-80E). The y-axis shows the binding of resonance units (RU), and the x-axis shows the elapsed time (second, s). The antibodies were serially diluted 2-fold down with concentrations of 200–6.25 nM. The kinetic analysis was performed at a panel of different concentration of the antibodies with a contact time of 300s followed by 300s dissociation. All measurements were performed at least three times. Data were analyzed by using BIA evaluation software 4.1, and fitted to 1:1 Langmuir binding model. The antibodies are d182, d448, d462, d511, d559, d622, and d628 as indicated. (TIF) [file ppat.1007716.s003.tif]

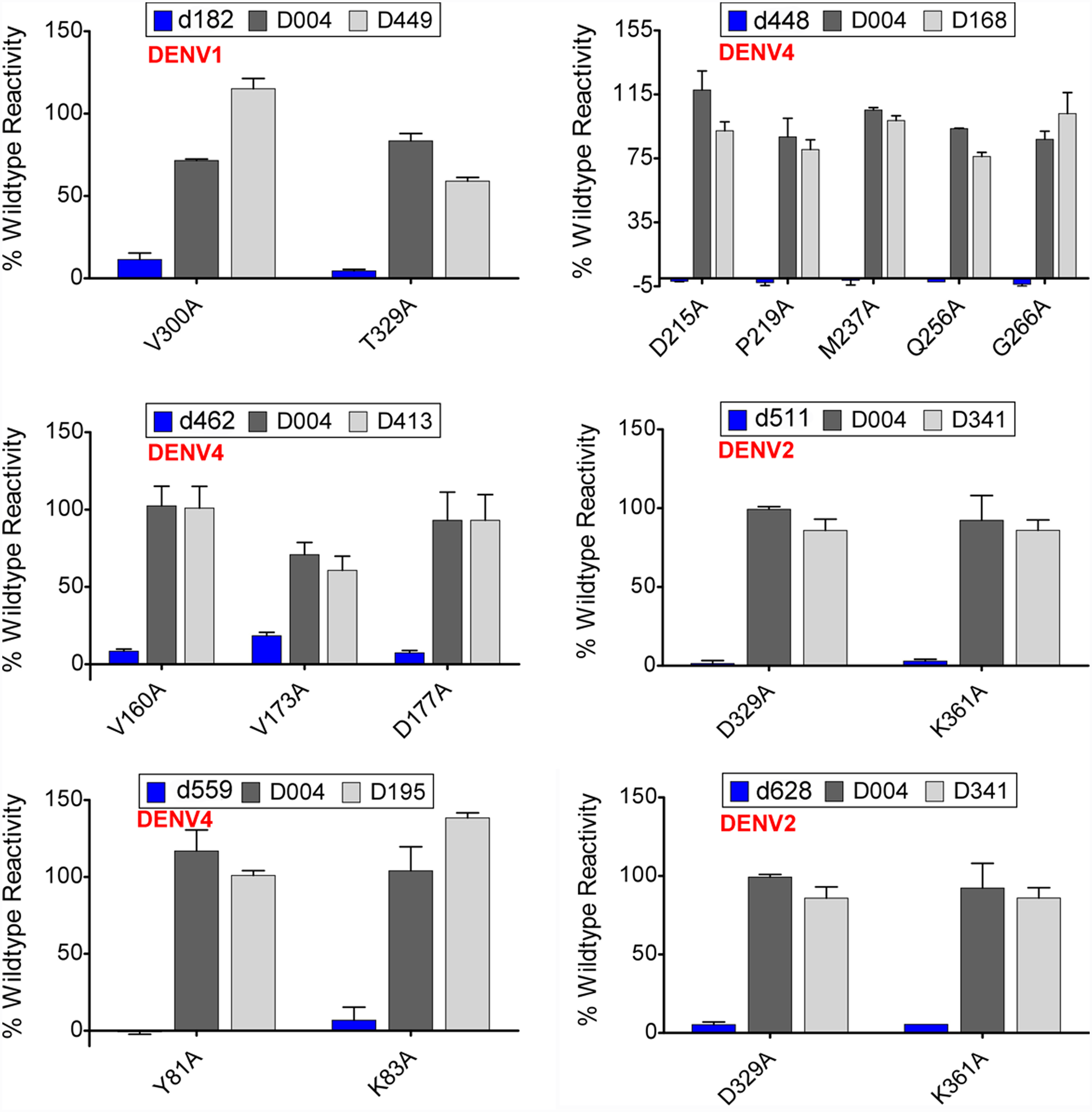

Supplement: S4 Fig — The ‘Shotgun Mutagenesis’ epitope mapping was performed by subjecting expression constructs for prM/E from all four DENV serotypes to high-throughput mutagenesis to generate comprehensive mutation libraries. The library consists of random mutations (to alanine, and analnie to serine) introduced at each residue of the prM/E polyprotein, while for DENV1 (strain WestPac), DENV2 (strain 16681), DENV3 (strain CH53489), and DENV4 (strain 341750). For screening, the DENV prM/E library was expressed in HEK-293T cells and assayed by immunofluorescence for the testing mAb binding to each clone. The antibody reactivity against each mutant protein clone was calculated relative to wild-type protein reactivity by subtracting the signal from mock-transfected controls (% Wildtype Reactivity). The mAb d182 (A) was mapped on the DENV1 library, d511 (D), d559 (E) and d628 (F) on the DENV2 library, and d462 (C), d448 (B), on the DENV4 library. The positive binding antibodies are D004, D449, D168, D413, D341, and D195 as indicated. Error bars represent the average of three measurements ± SD. (TIF) [file ppat.1007716.s004.tif]

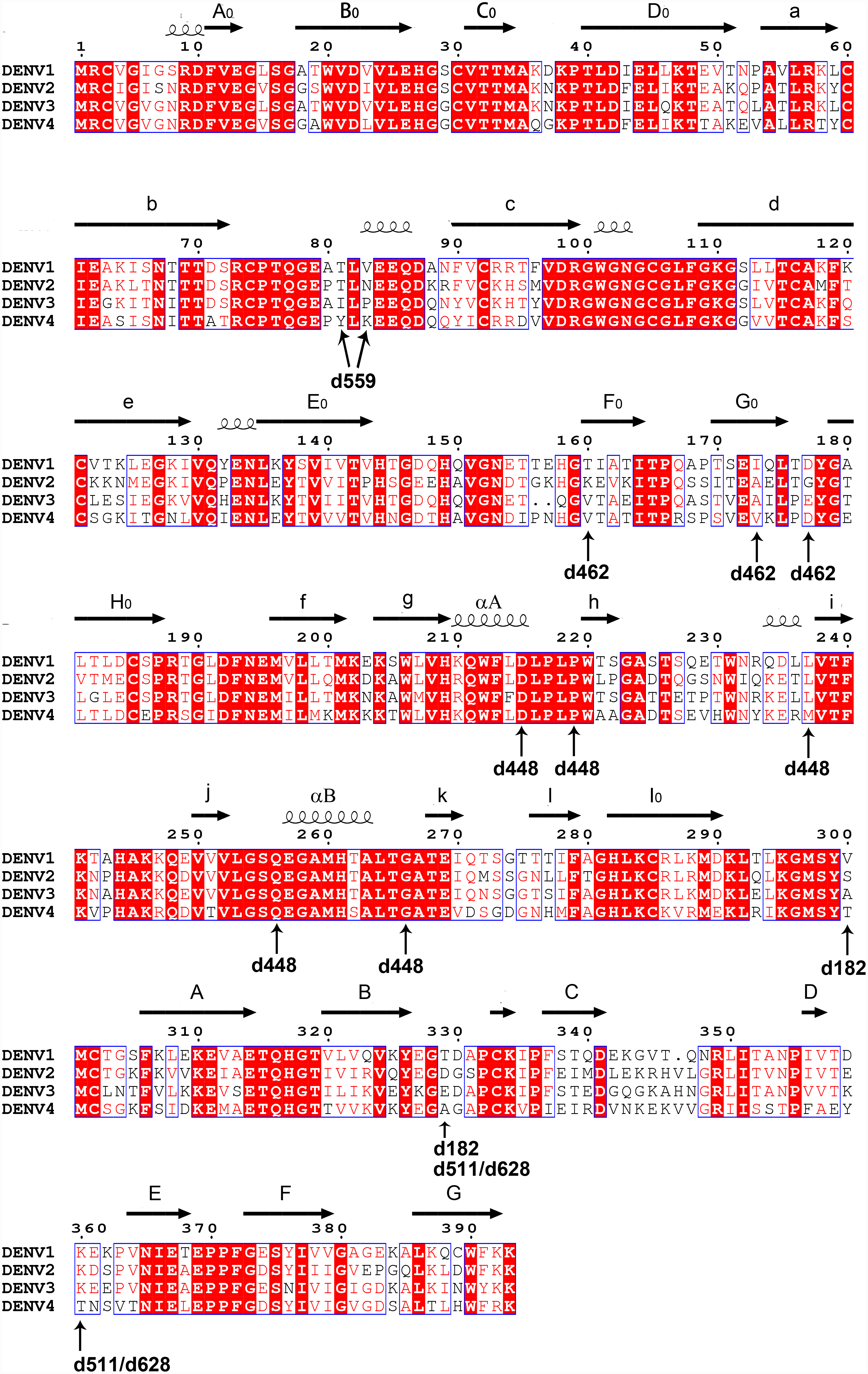

Supplement: S5 Fig — The envelope protein sequence alignment of DEN-80E of DENV1, DENV2, DENV3, and DENV4 was generated and analyzed using ClustalW2 (http://www.ebi.ac.uk/Tools/msa/clustalw2/) and ESPript 3.x (http://espript.ibcp.fr/ESPript/ESPript/). The NCBI accession numbers are ACJ04226 (DENV1), AGS49173 (DENV2), AJA37731 (DENV3) and ACW82884 (DENV4) respectively. Identical residues are shown as white text on a red background, and similar residues are shown as red text. The secondary structure of DEN-80E (PDB: 1OK8) are displayed above the residue numbers. The β-strands are labeled with different letters, as A0 to I0 for the domain I, a to l for domain II, and A to G for domain III. The residues on the epitope are marked with the indicated antibody under the alignment, respectively. (TIF) [file ppat.1007716.s005.tif]

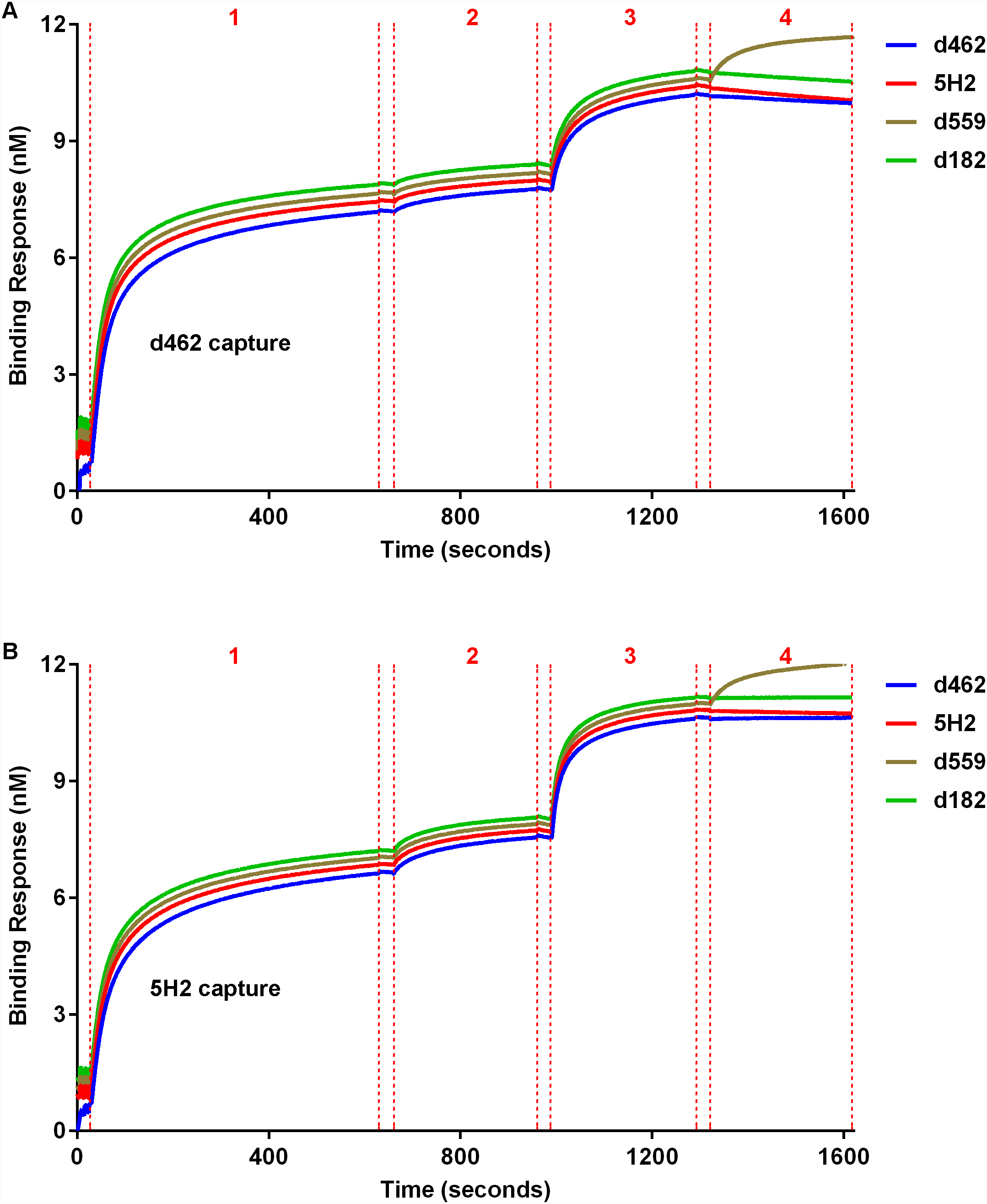

Supplement: S6 Fig — The real-time competitive binding of d462 and 5H2 was determined using an epitope binning format on Octet RED96 biosensor. The mAb d462 (A) or 5H2 (B) was first captured using protein A biosensor (step 1), followed by a blocking antibody to saturate the unoccupied sensors (step 2). The DEN4-80E was incubated to bind to the captured antibody (step 3). Finally, a secondary antibody was allowed to bind to the DEN4-80 on the sensors (step4). The binding of the secondary antibody will be blocked if it binds to the overlapped epitope with the captured antibody. An isotype control monoclonal antibody d182 was used as negative control (green line). The DENV4 specific mAb d559 was used as positive control (brown line). The d462 (blue line), 5H2 (red line) bind to DEN4-80E competitively. (TIF) [file ppat.1007716.s006.tif]

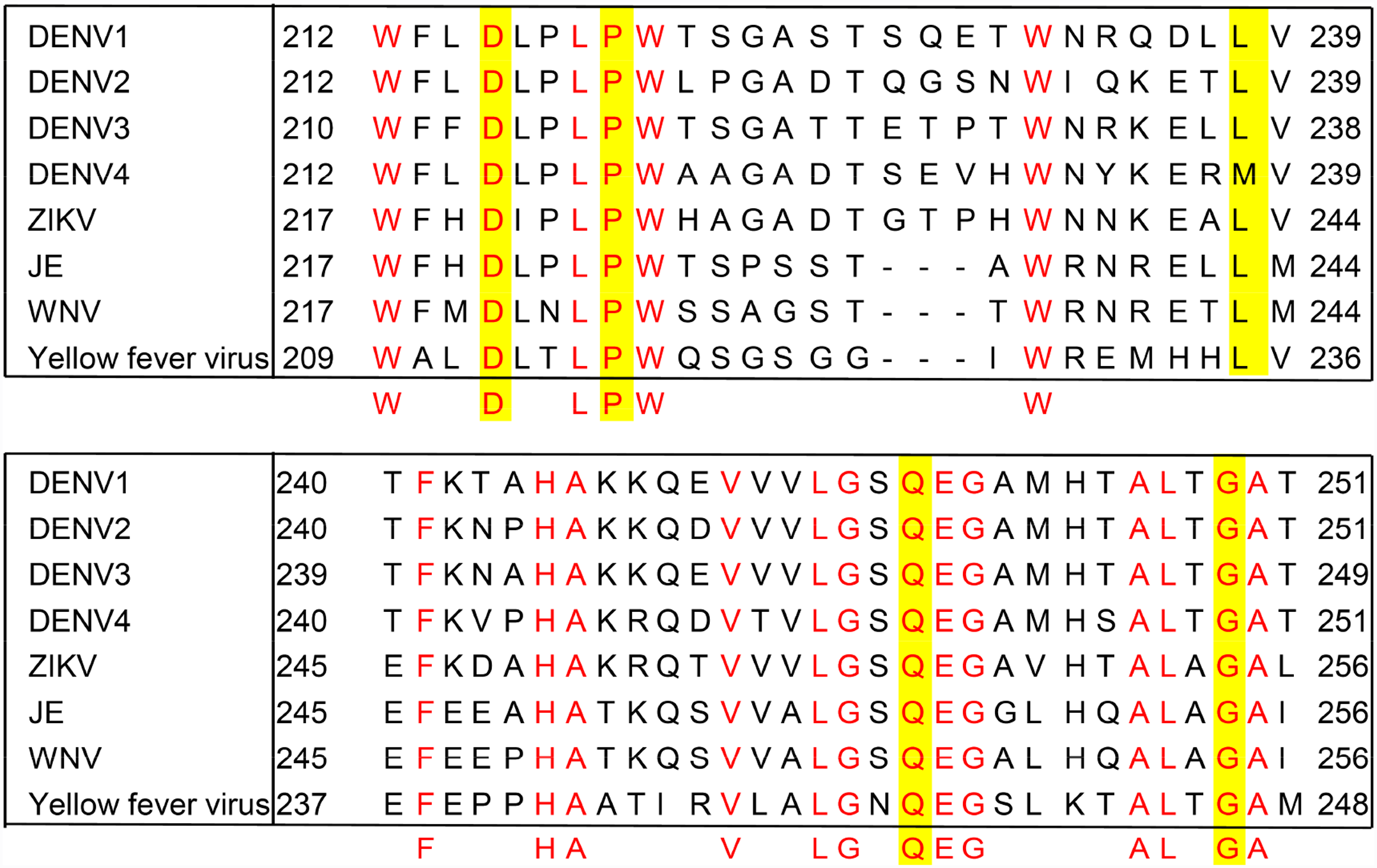

Supplement: S7 Fig — E proteins of Dengue 1 (DENV1), DENV2, DENV3, DENV4, ZIKA virus, Japanese encephalitis virus (JE), West Nile virus (WNV), and the yellow fever virus were aligned. Amino acids in red are those conserved among the flavivirus family. Residues highlighted in yellow are those on the epitope of the antibody d448. The GeneBank accession number of the sequences are: ACJ04226 (DENV1), AGS49173 (DENV2), AJA37731 (DENV3), ACW82884 (DENV4), ARK18853 (ZIKA), AFO42844 (JE), ACI95758 (WNV), and AAA92702 (yellow fever virus). (TIF) [file ppat.1007716.s007.tif]

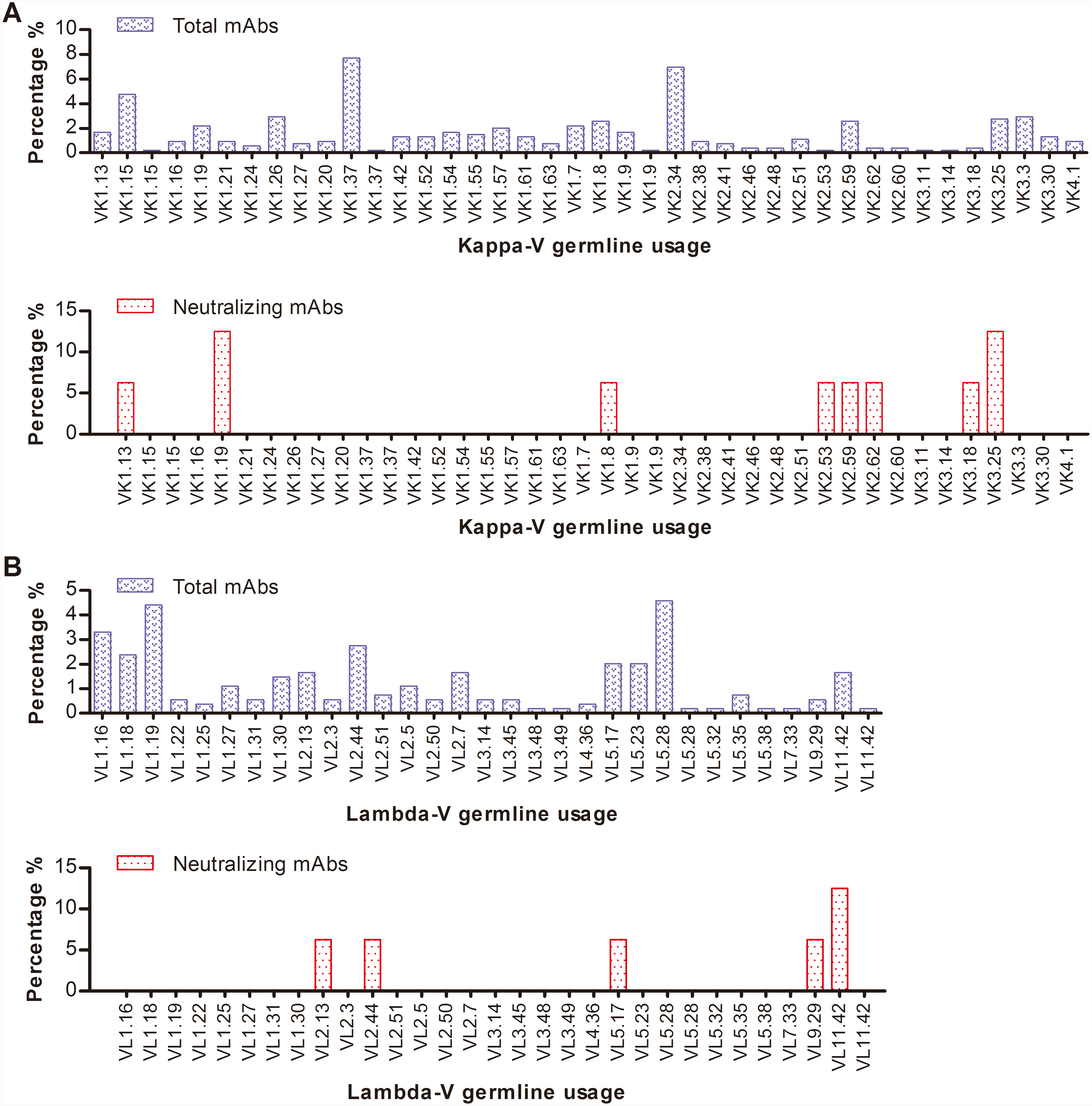

Supplement: S8 Fig — General light chain germline usage of the rhesus macaque antibodies. The analysis was performed using IgBLAST with IMGT V domain delineation (https://www.ncbi.nlm.nih.gov/igblast/). The entire panel contains all isolated mAbs and neutralizing mAbs. (A) The kappa chain V germline distribution of the cloned antibodies (blue), the neutralizing antibodies (red). (B) The lambda chain V germline distribution of the cloned antibodies (blue), the neutralizing antibodies (red). (TIF) [file ppat.1007716.s008.tif]
