# Supplementary material for: Potent neutralizing antibodies elicited by dengue vaccine in rhesus macaque target diverse epitopes
Source: PLoS Pathog. 2019 Jun 6;15(6):e1007716. doi: 10.1371/journal.ppat.1007716 (PMC6553876; doi:10.1371/journal.ppat.1007716)
Supplement: S1 Table — (DOCX) [file ppat.1007716.s009.docx]

**S1 Table. Relative titer of the reporter virus particles with residue substitution**

| RVP | Serotype | mAb | Relative titer compares to the wild type (WT) RVP (%) |
| --- | --- | --- | --- |
| DENV1 | | | |
| WT | 1 |  | 100.0 |
| V310A | 1 | d182 | 5.8 |
| V310G | 1 | d182 | 7.6 |
| T329A | 1 | d182 | 7.9 |
| T329G | 1 | d182 | 43.2 |
| DENV2 | | | |
| WT | 2 |  | 100.0 |
| D329G | 2 | d511/d628 | 4949.2 |
| D329E | 2 | d511/d628 | 102.5 |
| K361G | 2 | d511/d628 | 6467.7 |
| K361R | 2 | d511/d628 | 4700.8 |
| D215A | 2 | d448 | 11.4 |
| P219A | 2 | d448 | 13.4 |
| L237A | 2 | d448 | 40.3 |
| Q256A | 2 | d448 | n.a. |
| G266A | 2 | d448 | n.a. |
| DENV4 | | | |
| WT | 4 |  | 100.0 |
| Y81G | 4 | d559 | 10.8 |
| Y81T | 4 | d559 | n.a. |
| K83G | 4 | d559 | 21.1 |
| K83R | 4 | d559 | 101.0 |
| V160A | 4 | d462 | 4.8 |
| V160G | 4 | d462 | 398.4 |
| D173A | 4 | d462 | 405.0 |
| D173G | 4 | d462 | 25.9 |
| D177A | 4 | d462 | n.a. |
| D177G | 4 | d462 | 6.6 |
| D215A | 4 | d448 | n.a. |
| P219A | 4 | d448 | 19.9 |
| M237A | 4 | d448 | 33.3 |
| Q256A | 4 | d448 | 6.0 |
| G266A | 4 | d448 | 8.7 |

The “n.a.” indicates no production of the reporter virus particle.
