# Supplementary material for: Potent neutralizing antibodies elicited by dengue vaccine in rhesus macaque target diverse epitopes
Source: PLoS Pathog. 2019 Jun 6;15(6):e1007716. doi: 10.1371/journal.ppat.1007716 (PMC6553876; doi:10.1371/journal.ppat.1007716)
Supplement: S2 Table — (DOCX) [file ppat.1007716.s010.docx]

**S2 Table. Germline usage of the potent neutralizing antibodies**

| mAbs | Isotype | Heavy | Light |
| --- | --- | --- | --- |
| d182 | *κ* | VH4.26 | VK3.18 |
| d511 | *λ* | VH4.57 | VL11.42 |
| d628 | *λ* | VH4.22 | VL11.42 |
| d559 | *λ* | VH4.38 | VL2.13 |
| d462 | *κ* | VH4.26 | VK1.8 |
| d448 | *κ* | VH4.34 | VK3.25 |
